# FAT1 Upregulates in Oral Squamous Cell Carcinoma and Promotes Cell Proliferation *via* Cell Cycle and DNA Repair

**DOI:** 10.3389/fonc.2022.870055

**Published:** 2022-05-11

**Authors:** Ting Lan, Qi Ge, Ke Zheng, Li Huang, Yuxiang Yan, Lixin Zheng, Youguang Lu, Dali Zheng

**Affiliations:** ^1^ Fujian Key Laboratory of Oral Diseases, Fujian Biological Materials Engineering and Technology Center of Stomatology, School and Hospital of Stomatology, Fujian Medical University, Fuzhou, China; ^2^ Department of Pathology, The First Affiliated Hospital of Fujian Medical University, Fuzhou, China; ^3^ Department of Dentistry, The First Affiliated Hospital of Fujian Medical University, Fuzhou, China; ^4^ School of Mathematics, Georgia Institute of Technology, Atlanta, GA, United States; ^5^ Department of Preventive Dentistry, School and Hospital of Stomatology, Fujian Medical University, Fuzhou, China

**Keywords:** Fat1, OSCC, cell proliferation, TCGA, RNA-seq

## Abstract

**Objective:**

Previous studies have revealed that FAT atypical cadherin 1 (FAT1) plays a tumor-suppressive or oncogenic role in a context-dependent manner in various cancers. However, the functions of FAT1 are ambiguous in tumorigenesis owing to inconsistent research in oral squamous cell carcinoma (OSCC). The present study aimed at gaining an insight into the role of FAT1 in the tumor genesis and development.

**Methods:**

The expression, mutant, and survival data analyses were done using data from The Cancer Genome Atlas (TCGA), the Gene Expression Omnibus (GEO), and the Clinical Proteomic Tumor Analysis Consortium (CPTAC) database, verified with clinical samples *via* real-time polymerase chain reaction (qRT-PCR), Western blot (WB), and immunohistochemical (IHC) staining. OSCC cells transfected with siRNA were employed for *in vitro* assessment in cell proliferation, apoptosis, and migration ability in appropriate ways. The underlying mechanism was explored by RNA sequencing after FAT1 silencing.

**Results:**

Overall, FAT1 significantly increased in OSCC with a poor prognosis outcome. The *in vitro* experiment showed the promoting effect of FAT1 in the proliferation and migration of OSCC cells. FAT1 can also inhibit both the early and late apoptosis of OSCC cells. RNA-sequencing analysis of FAT1 silencing revealed that the cell cycle, DNA replication, and some core genes (MCM2, MCM5, CCNE1 SPC24, MYBL2, KIF2C) may be the potential mechanism in OSCC.

**Conclusions:**

FAT1 may act as an oncogene in OSCC with potential mechanism influencing the cell cycle and DNA repair.

## Introduction

Oral squamous cell carcinoma (OSCC), a subset of head and neck squamous cell carcinoma (HNSC), is always lethal. Mainly based on stage and anatomic location, the current standard therapy for OSCC consists of surgery and radiation therapy, which are generally recommended for the approximately 30% to 40% of individuals in the early-stage disease (stage I or II) (1). Like all solid tumors, OSCCs are thought to be initiated through a series of genetic alterations (2). Integrated genomic analysis has identified FAT1 as an additional driver gene, which has been detected mutant in several large-scale exome sequencing projects, frequently in esophageal squamous cell carcinoma (ESCC) and OSCC (3, 4). FAT1 (FAT atypical cadherin 1) is a member of the vertebrate Fat cadherin family, which comprised FAT1, FAT2, FAT3, and FAT4 genes (5). First isolated from the T-leukemia cell line J6, FAT1 is located on human chromosome 4q35.2, consists of 27 exons, and encodes proteins with a single transmembrane domain, 34 extracellular cadherin repeats, and laminin G-like and epidermal growth factor (EGF)-like domains (5, 6). FAT1 is widely expressed in many fetal tissues whereas it is downregulated or disappears in most adult tissues, indicating that FAT1 may play a role in development (7).

However, compared to the ample studies and information available of FAT1 over the past two decades, the role of FAT1 in tumor initiation and progression has been conflicting. Morris et al. identified FAT1 as a candidate tumor-suppressor gene which is able to suppress cancer cell growth by binding β-catenin and antagonizing its nuclear localization in glioblastoma (GBM) (8). Similarly, in breast cancer, it has been reported that the loss of FAT1 was associated with progression, aggressive behavior, poor prognosis, and cyclin-dependent kinase (CDK) 4/6 inhibitor resistance through the Hippo signaling pathway (9, 10). The expression of FAT1, as the transcriptional target of E2F1, was frequently downregulated in ESCC tissues and inhibited proliferation, adhesion, and invasion through the MAPK signaling pathway (11, 12). Yu et al. suggested that a low FAT1 expression was associated with poor prognosis in children with medulloblastoma and acted on the WNT signaling pathway to inhibit cell proliferation (13). On the other hand, FAT1 functions as an oncogene in many other cancers. FAT1 was shown to be aberrantly expressed in pediatric patients with acute leukemia, whereas hematopoietic progenitors from healthy donors lacked the FAT1 expression. FAT1 expression was also correlated with a more mature leukemic immunophenotype (14, 15). Furthermore, FAT1, reported as a new glypican-3 (GPC3)-interacting protein, appeared as a relevant mediator of hypoxia and growth receptor signaling to critical tumorigenic pathways with a higher expression in HCC (16, 17).

In regard to head and neck cancers, Lin et al. reported that a lower FAT1 expression was correlated with poor disease-free survival, and they proved that FAT1 suppressed the migration and invasion capability of the SCC25, FaDu, HSC3, OECM-1, and OC4 HNSC cell lines, not changing the cell proliferation (18). Martin et al. supported that the FAT1 intracellular domain (ICD) interacted with and facilitated the assembly of the core Hippo signaling complex as upstream of Yes-associated protein 1 (YAP1). Inactivation mutations and genomic alterations in FAT1 resulted in HNSC by activating YAP1 to suppress the HIPPO signaling pathway and promoted the proliferation (19). Interestingly, Hsu et al.’s findings contradicted those of Lin et al., and they cautiously attributed to tumor heterogeneity and/or cohort constitution, similar to the studies of GBM in which the expression level depended on the grade of cancerous cells (20–22).

In this study, we aimed to comprehensively analyze the expression of FAT1 and its effect on OSCC as well as some exploration of mutations which may cause gene expression changes. In addition, we silenced FAT1 to evaluate the effects on cell death and survival, proliferation, and migration in two OSCC cells. Finally, RNA sequencing (RNA-seq) was performed to investigate the gene expression profile upon FAT1 knockdown. FAT1 was thus considered to be a potential target for the development of molecular therapeutic strategies to improve the prognosis of OSCC.

## Materials and Methods

### Data Preparation From Public Database and Statistical Analysis

RNA-seq (HTSeq-FPKM), mutation, and corresponding clinical data were downloaded from The Cancer Genome Atlas (TCGA) HNSC project in March 2020 using the R package TCGAbiolinks (23). The transcriptome data were log10-transformed for comparison. The R package maftools was utilized to analyze and visualize the somatic condition of HNSC patients from TCGA (24). The GSE6631, GSE37991, GSE30784, and GSE10300 datasets were retrieved from the Gene Expression Omnibus (GEO) database (https://www.ncbi.nlm.nih.gov/geo/). Raw data were examined within normalization and log2 transformation, and then the gene probes were annotated to explore the FAT1 expression level. Kaplan–Meier (KM) survival analysis was undertaken to compare the overall survival of different groups of patients *via* the R package survival and survminer (determined the optimal cutpoint), and P-values were calculated using the log-rank test. The COSMIC database (https://cancer.sanger.ac.uk/cosmic) was used to determine the occurrence of FAT1 mutation variants.

Processed mass spectrometry data of HNSC were downloaded from the Clinical Proteomic Tumor Analysis Consortium (CPTAC) Data Portal (https://cptac-data-portal.georgetown.edu/), a 108 human papilloma virus (HPV)-negative HNSC cohort. After plus 10, raw data were converted into log10. Data used in this publication were generated by the Clinical Proteomic Tumor Analysis Consortium (NCI/NIH).

All statistical analyses and visualizations were performed using R (version 4.0.1) or GraphPad Prism (version 6.01). For comparisons, Student’s t-test, Mann–Whitney U-test, Fisher’s exact test, or chi-square test was used when appropriate and P < 0.05 was considered statistically significant for all tests.

### Clinical HNSC Specimens

This study was approved by the Institutional Review Board of Fujian Medical University School and Hospital of Stomatology (Approval Number: FMUSS-18-004). Samples and clinical information were collected as described (25). The patient clinicopathologic characteristics included age, sex, tumor stage, differentiation degree, metastasis status, depth of invasion, extra extension, and perineural invasion. Patient death was mainly caused by carcinoma recurrence or metastasis. Another 33 paired fresh samples were collected to quantify the FAT1 mRNA expression, and we confirmed 8 pairs at the protein level.

### Total RNA Isolation and Quantitative Reverse Transcription Polymerase Chain Reaction

Total RNA was extracted from tissue samples using NucleoZOL Reagent (Takara, Dalian, China) according to the manufacturer’s protocol, and cDNA was synthesized from 1 µg of total RNA using a PrimeScript^®^ 1st strand cDNA Synthesis Kit (Takara, Dalian, China) in a final reaction volume of 20 µl. Quantitative RT-PCR was performed using SYBR Premix Ex Taq (Takara, Dalian, China) with primers listed in **Table 1**. The expression levels were normalized to the GAPDH mRNA level for each sample obtained from parallel assays, and the data were analyzed according to the relative 2^ (-ΔΔCt) method.

**Table 1:** The primers for qRT-PCR in the current study.

Gene	Forward	Reverse
GAPDH	5′-GGTGTGAACCATGAGAAGTATGA-3′	5′-GAGTCCTTCCACGATACCAAAG-3′
FAT1	5′-GCACCTGTTGGTTCATTGGTAA-3′	5′-AATAATGGGAGGTCGATTCACG-3′
MCM2	5′-ACCCGAAGCTCAACCAGATG-3′	5′-ATAGTCCCGCAGATGGATGC-3′
MCM5	5′-TCGTCAAGGATGAGCACAATG-3′	5′-TCACTCGGCAGTAGGCAATA-3′
CCNE1	5′-CTGGATGTTGACTGCCTTGAAT-3′	5′-TCTCTATGTCGCACCACTGAT-3′
CD83	5′-CTGCTGCTGGCTCTGGTTAT-3′	5′-CAGTTCTGTCTTGTGAGGAGTCA-3′
SPC24	5′-GCTGCGAGAGATCCTCACCAT-3′	5′-TGGCCTTCAGACGGGTGT-3′
MYBL2	5′-GCTGGCATCGAACTCATCAT-3′	5′-GCTTCACATCCTCATCCACAAT-3′
KIF2C	5′-GATGGAAGCCTGCTCTAACG-3′	5′-AGTCTGGTCCTTGCTGTATGA-3′
SUV39H1	5′-GTGGATGCCGCCTACTATGG-3′	5′-CGCTCGTCAAGGTTGTCTATG-3′
UHRF1	5′-CCAACCACTACGGACCCATC-3′	5′-ACTAGGGAGTACGCTCCGTC-3′
PERP	5′-TACTCAGCGCCATCGCCTTC-3′	5′-TCTTGGGAGCATTTCCACCAC-3′
TPM4	5′-GGAAGAGGCTGACCGCAAAT-3′	5′-TTAGTTCAGACACCTCCGCAC-3′
GANAB	5′-CGGCGGTCTTCAGAATGTATG-3′	5′-TGTTGCCAGAGAATGAGAATCG-3′
EMC6	5′-CCTGGATTATTGCCGGACCTC-3′	5′-AGGCGAGCAGGTAGAAGATG-3′

### IHC Staining

The tissue microarrays and immunohistochemistry were performed as previously described (25). Antigen retrieval was performed with EDTA buffer (pH 8.0) using a high-temperature and high-pressure antigen repair method. The slides were incubated with a primary antibody, polyclonal rabbit anti-human FAT1 (1:500; ab190242, Abcam, Cambridge, MA, USA), for 3 h at 25°C. Secondary antibodies were applied for 20 min at 25°C. All cases were reviewed and scored independently by two senior pathologists without knowledge of the clinical characteristics. The immunoreactivity intensity was scored in the following four categories: 0 (no staining), 1+ (weak staining), 2+ (moderate staining), and 3+ (strong staining). The immunoscore was obtained by multiplying (3*x% + 2*x% + 1*x% = total score) the percentage of positively stained tumor cells (0%–100%) by the corresponding immunostaining intensity (0 to 3+) to obtain a value ranging from 0 to 300. An immunoscore of 150 was set as the cutoff point for negative or positive expression (26), while another cutoff value was used in subsequent survival analyses determined by the R package survminer.

### Western Blot Assay

Using RIPA (Beyotime, China), after specific treatments, HN6 and HN30 cells were collected and lysed. After the protein concentrations was determined by the BCA method (Beyotime, Shanghai, China), the lysates were diluted into 5× SDS buffer to a 1× SDS final concentration and then heated for 10 min at 100°C. Proteins were separated on 4%–12% SDS-PAGE gels and electrophoresed at 30-V constant voltage overnight to polyvinylidene fluoride (PVDF) membranes. Blots were blocked with 5% BSA in TBST at room temperature for 1 h and then incubated overnight at 4°C with a primary antibody against FAT1 (1:1,000; ab190242, Abcam) and actin (1:1,000; ab179467, Abcam), followed by a horseradish peroxidase–conjugated secondary antibody for 1 h at room temperature. The membranes were washed thrice with TBST. Finally, they were visualized with Pierce™ Enhanced Chemiluminescence (ECL) Western Blotting Substrate (Beyotime, China) and imaged with a densitometer for semi-quantification of the signal intensity and later analyzed with the NIH ImageJ software (https://imagej.nih.gov/ij/download.html). The experiment was repeated three times.

### Cell Lines and Culture

The HN6 and HN30 cell lines were bought from the Cell Bank of the Institute of Biochemistry and Cell Biology, Chinese Academy of Sciences (Shanghai, China). Cells were cultured under the conditions provided by the supplier.

### Small Interfering RNA Transfection

Small interfering RNA (siRNA) logos against human FAT1 (siFAT1-1, synthesized as follows: sense: 5′-GCACCACAAUUUCGAGCAATT-3′, antisense: 5′-UUGCUCGAAAUUGUGGUGCTT-3′; siFAT1-2, synthesized as follows: 5′-GCACGUGUGUUGUCGACAATT-3′, antisense: 5′-UUGUCGACAACACACGUGCTT-3′) and a scrambled siRNA used as negative control (NC, synthesized as follows: sense: 5′-UUCUCCGAACGUGUCACGUTT-3′, antisense: 5′-ACGUGACACGUUCGGAGAATT-3′) were purchased from GenePharma (GenePharma, Shanghai, China). Cells were transfected with siRNAs with Lipofectamine RNAiMAX (Cat. 13778075, Invitrogen, Carlsbad, CA, USA) according to the manufacturer’s instructions. On the following day, cells were harvested for quantitative reverse transcription polymerase chain reaction (qRT-PCR), Western blot analysis, and other assays.

### Cell Proliferation Analysis

After transfection for 24 h, cells were collected from the interference group and NC group and then reseeded in 96-well plates at a density of 4,000 cells/well with 3 replicate wells per group. Cell Counting Kit-8 (Dojindo, Kumamoto, Japan) was employed to quantitatively evaluate cell viability every 24 h for 6 days. The absorbance was measured at 450 nm in a microplate reader (Pharmacia Biotech, Piscataway, NJ, USA). The experiment was performed in triplicate and repeated three times.

### Clone Formation Assay

After transfection for 24 h, cells were collected from the interference group and NC group and then reseeded in 6-well plates with 4,000 cells per well. Three duplicate wells were performed for each group. The plates were incubated at 37°C for 14 days and stained with crystal violet. They were then air-dried, and the numbers of clones were calculated. The experiment was performed in triplicate and repeated three times.

### Cell Cycle Assay

After transfection for 48 h, cells from the gene silencing group and NC group were harvested and washed three times with precooled PBS and then treated with 70% ethanol for at 4°C at least overnight. Then the cells were washed three times with PBS and resuspended in 500 µl PI/RNase Staining Buffer (550825, BD Biosciences, Franklin Lakes, NJ, USA) according to the manufacturer’s instructions. Cell cycle status was tested using a BD Accuri C6 flow cytometer (BD Biosciences) and analyzed by FlowJo software. The experiment was repeated three times.

### Cell Apoptosis Analysis With Annexin V-FITC/PI

After transfection for 48 h, cells were collected by trypsinization and centrifuged with supernatant at 1,500×g for 5 min. After being resuspended and incubated at 37°C for 30 min, the cells were washed three times with 4°C PBS and resuspended in 100 µl 1× binding buffer. 5 µl Annexin V-FITC and 5 µl propidium iodide (PI) solution (YF^®^ 488 Annexin V and PI Apoptosis Kit, US Everbright, Suzhou, China) were then added to stain the cells under darkness for 15 min. The apoptosis rate was measured by a BD Accuri C6 flow cytometer (BD Biosciences). Single-stained and unstained cells were used as a control. The experiment was repeated three times.

### The Sphere-Forming Assay

After transfection for 24 h, cells were dissociated to produce single-cell suspensions and were seeded in 96-well ultralow-attachment plates (Corning, Tewksbury, MA, USA) at a density of 800 cells/well. They were cultured in mTeSR medium (STEMCELL Technologies, Vancouver, Canada). After incubation for 1–2 weeks, tumorspheres were photographed under a microscope (×50 magnification) and the number was determined. The experiment was performed in triplicate and repeated three times.

### Migration Assay

After transfection for 24 h, using a 24-well plate, we seeded 5 × 10^4^ cells for HN6 and 3 × 10^5^ cells for HN30 into the upper chamber of the insert (8-μm pore size, BD Biosciences, USA) containing FBS-free media, while the lower chamber contained 10% FBS-supplemented media. After incubation for 24 h for HN6 and 48 h for HN30, the chambers were stained with crystal violet and non-invaded cells in the upper surface were removed with sterile cotton swipes. Then, the average number of invaded cells was determined and photographed under a microscope (×200 magnification), from at least five non-overlapping visual fields selected randomly. The experiment was repeated three times.

### Wound Healing Assay

After transfection for 24 h, cells were reseeded in 12-well plates and until cell monolayers were cultured, scratched by manually scraping off cells with a sterile yellow pipette tip. All the wound sizes were verified to be of similar width in the beginning. The images of cell migration were observed and captured at the 18- and 32-h time-points afterward under a ×50 microscope magnification (Olympus, Tokyo, Japan) and later analyzed with the NIH ImageJ software (https://imagej.nih.gov/ij/download.html). The experiment was repeated three times.

### RNA Sequencing and Analysis

After transfection for 24 h, cells were harvested and the total RNA extracted using NucleoZOL Reagent (Takara, Dalian, China) according to the manufacturer’s protocol. RNA-sequencing libraries were constructed with FAT1 knockdown (siFAT1-1 and siFAT1-2) and the control group in two HN6 and HN30, repeated two biological times. RNA-seq was performed by Berry Genomics company. Briefly, RNA integrity was evaluated using NanoDrop 2000 or an Agilent 2100 Bioanalyzer. All included samples had RNA concentrations >40 ng/µl, and a total amount of >2 µg with an RNA integrity number (RIN) ≥6.5 was subjected to subsequent analyses. The library preparations were sequenced on an Illumina NovaSeq 6000 platform, and 150-bp paired-end reads were generated. Raw reads were processed using custom scripts, and ploy-N-containing reads, PCR duplications, and low-quality reads were removed to obtain clean reads, which were then mapped to the hg38 genome. R package Limma-voom was used for the differential expression analysis. Abs (log_2_ fold change) >1.00 and P-value <0.05 were used as criteria to classify differentially expressed genes (DEGs) (27). Gene Set Enrichment Analysis (GSEA) based on the ordered list of all genes according to the log2 fold change (log2FC) value, Gene Ontology (GO) functional enrichment analyses, and Kyoto Encyclopedia of Genes and Genomes (KEGG) pathway analyses were performed by the R package clusterProfiler (28). Cancer hallmark definitions were downloaded from GSEA/MSigDB 6.2 (http://www.broadinstitute.org/gsea/msigdb). The STRING database (https://string-db.org/) was used to produce protein–protein interaction networks (PPI) and the enrichment analyses based on DEGs, and the networks was visualized *via* Cytoscape 3.9. P-value < 0.05 was set as a significant enrichment criterion.

## Results

### High FAT1 mRNA Expression Level in OSCC Correlates With Poor Prognosis

**Figure 1:**
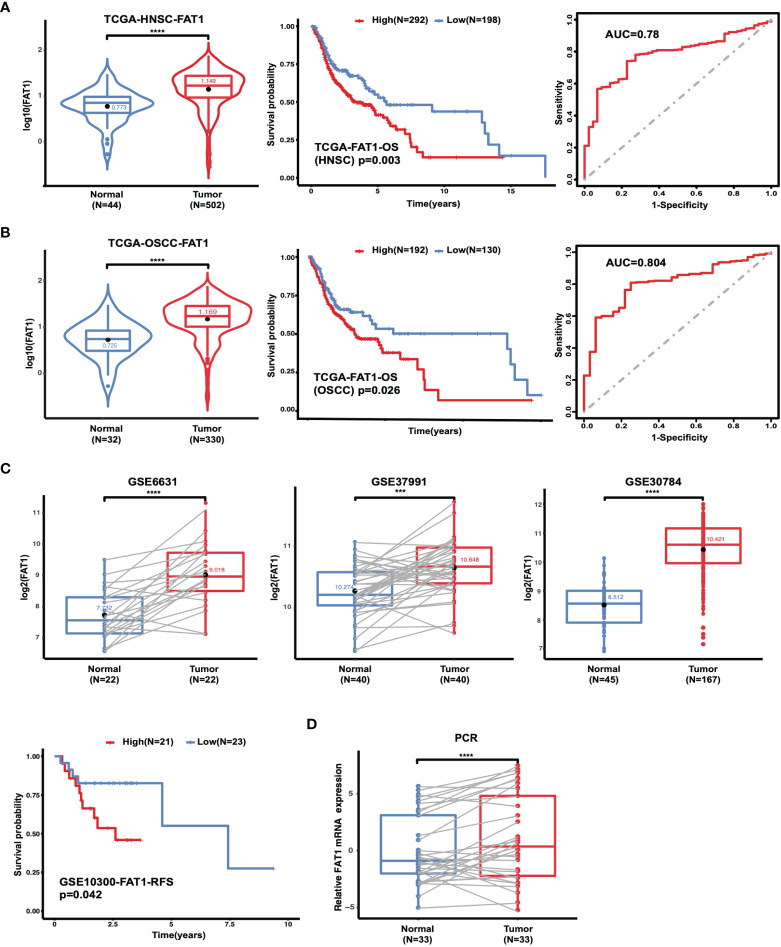
The FAT1 mRNA level is upregulated in HNSC and OSCC, and a higher FAT1 expression level is associated with shorter overall survival. **(A)** FAT1 mRNA expression status between normal tissues and tumor tissues in HNSC from TCGA (boxplot and violin plot); Kaplan–Meier survival curve of overall survival time in HNSC from TCGA; areas under the curve (AUCs) of the ability to discriminate normal tissues and tumor tissues by the FAT1 mRNA level in HNSC from TCGA. **(B)** FAT1 mRNA expression status between normal tissues and tumor tissues in OSCC from TCGA (boxplot and violin plot); Kaplan–Meier survival curve of overall survival time in OSCC from TCGA; areas under the curve (AUCs) of the ability to discriminate normal tissues and tumor tissues by FAT1 mRNA level in OSCC from TCGA. OSCC is the main subtype of HNSC (including the sites of mouth, cheek mucosa, tongue, hard plate, lip, and gum). **(C)** FAT1 mRNA expression status in paired tumor and non-tumor pair-wise data sources (GSE6631 and GSE37991) and another GSE30784 set (boxplot). Kaplan–Meier survival curve of recurrence-free survival time in HNSC from GSE10300. **(D)** FAT1 mRNA expression status in clinical samples (boxplot). ***P < 0.001; ****P < 0.0001.

Because of the controversial roles of FAT1 reported in different malignancies also in HNSC, we first evaluated in TCGA database to figure out its expression pattern. Data from HNSC samples (n = 502) revealed a significantly higher expression level of FAT1 in comparison with the adjacent normal tissues (n = 44; P < 0.0001). According to the Kaplan–Meier survival analysis, the survival rate was dramatically lower in FAT1 high expression group patients (P = 0.003). The results between OSCC samples (n = 330, the main subtype of HNSC) and adjacent normal tissues (n = 32) showed the same trend. Furthermore, FAT1 exhibited moderate classification ability between the tumor tissues and normal tissues lesion types with an AUC = 0.78 in HNSC and an AUC = 0.804 in OSCC (**Figures 1A, B**). Together, these results provided important insights into the potential capacity of FAT1 as a biomarker. Then, we analyzed further data from more than one region derived from the GEO database. Also, FAT1 was upregulated in tumor samples from two independent tumor and non-tumor pair-wise data sources (GSE6631 with HNSCs and GSE37991 with OSCCs; P < 0.0001, P < 0.001) and another set with 167 OSCCs and 45 normal oral tissues (GSE30784; P < 0.0001). Through the GEO cohort (GSE10300 with HNSC) with clinical data (recurrence-free survival), we could see that a higher expression of FAT1 based on the median was associated with a poor overall survival (P = 0.042 in RFS, **Figure 1C**). We also validated the FAT1 expression level with RNAs extracted from OSCC tissues and their matched normal tissues by quantitative real-time PCR (**Figure 1D**). Consistently, they showed statistically higher expression levels of FAT1 (P < 0.0001). Taken together, our findings demonstrated that a high FAT1 expression occurred in HNSC and OSCC, which was associated with poor patient survival and may play an oncogenic role in OSCC.

### High FAT1 Protein Expression Level in OSCC Associated With Adverse Clinicopathological Features

**Figure 2:**
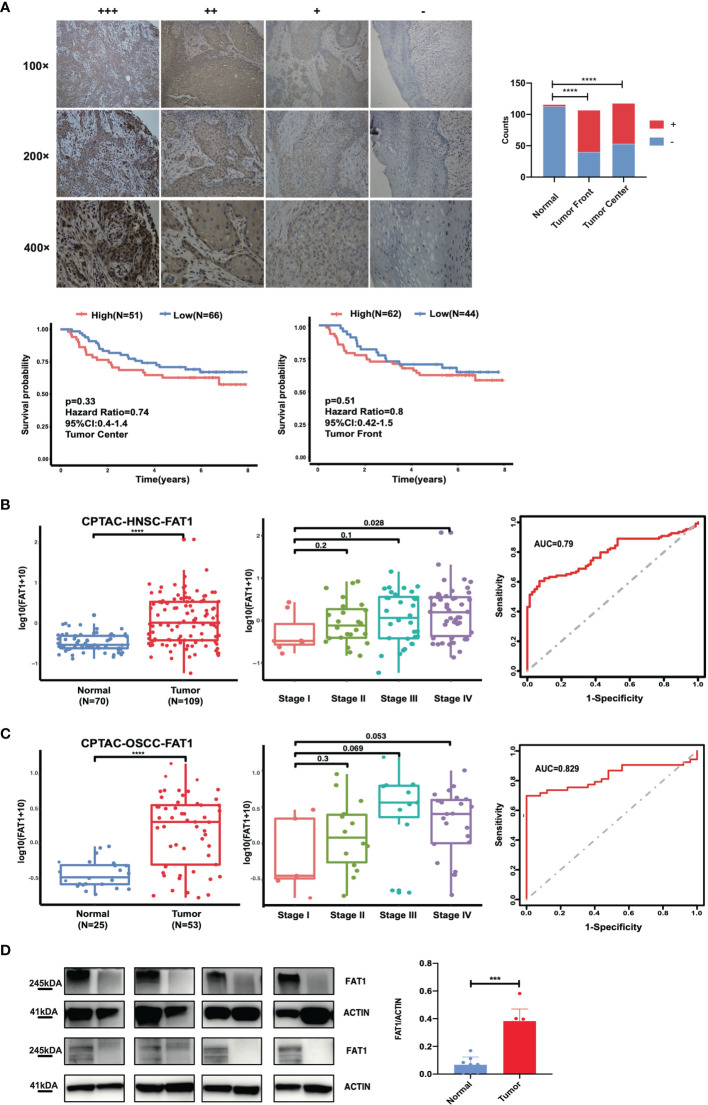
FAT1 protein levels are upregulated in HNSC and OSCC tissues. **(A)** Left: Representative immunohistochemical staining images of normal and tumor tissues (magnification at ×100, ×200, ×400). FAT1 localization appears as brown staining in the cytoplasm. Right: histogram of the proportion of the FAT4 staining levels among the tumor front, tumor center, and adjacent normal samples and Kaplan–Meier survival analysis of patients with high and low levels of FAT1 protein expression by immunohistochemical staining levels. A high FAT1 expression exhibited poor survival but not significantly (P > 0.05). **(B)** FAT1 protein expression status between normal tissues and tumor tissues in HNSC from CPTAC (boxplot); FAT1 protein expression status among high stage and stage I tumor tissues in HNSC from CPTAC (boxplot); areas under the curve (AUCs) of the ability to discriminate normal tissues and tumor tissues by the FAT1 protein level in HNSC from CPTAC. **(C)** FAT1 protein expression status between normal tissues and tumor tissues in OSCC from CPTAC (boxplot); FAT1 protein expression status among high stage and stage I tumor tissues in OSCC from CPTAC (boxplot); areas under the curve (AUCs) of the ability to discriminate normal tissues and tumor tissues by the FAT1 protein level in OSCC from CPTAC. **(D)** FAT1 protein expression status in clinical samples (bar plot). ***P < 0.001; ****P < 0.0001.

To study the clinical significance and protein expression level of FAT1 in OSCC, we next examined 122 tissues by IHC. The expression levels of FAT1 were evaluated independently at the tumor center and the invasion front, which was defined as the tumoral advancing edge. FAT1 gave a positive staining in 62.6% (67/107) tumor front samples, 55.1% (65/118) tumor center samples, and 0.3% (3/116) normal tissues. FAT1 was detected mainly in the cytoplasm of squamous carcinoma cell (weak staining in the region of squamous pearl formation), and normal oral mucosa specimens displayed positivity mainly in sporadic cells of basal layers. The proportion of FAT1-positive tumor front and tumor center samples was significantly higher than that in non-cancerous samples (P < 0.0001). Kaplan–Meier survival analysis demonstrated that a high expression of FAT1 indicated a poor prognosis of OSCC, but the differences did not reach statistical significance (P = 0.33 in tumor center, P = 0.51 in tumor front; **Figure 2A**). The FAT1 expression of tumor front was significantly or nearly related with age (P = 0.0379), metastasis (P = 0.0063), tumor bunding (P = 0.0168), and perineural invasion (P = 0.0535) while the FAT1 expression of tumor center was significantly or nearly related with age (P = 0.0114) and differentiation (P = 0.0203) and metastasis (P = 0.0532). Statistical significance was not found in gender, tumor stage, depth of invasion (DOI), or extranodal extension (ENE) (**Table 2**). What emerged from the IHC results here was that FAT1 exhibited a high expression localized in the cytoplasm and was associated with the adverse clinicopathological features in OSCC. In addition, protein expression levels of FAT1 were determined in a cohort of 109 head and neck tumors and 70 normal head and neck tissue samples in the CPTAC database, consistently, which was upregulated in HNSC tissues (P < 0.0001). Moreover, we observed the FAT1 expression presenting an elevated trend in progressing tumor stages but not significantly. Interestingly, the FAT1 expression significantly increased in stage IV compared with that in low disease stages (P < 0.05). The results between OSCC samples (including lips and oral cavity, n = 53, a main subtype of HNSC) and adjacent normal tissues (n = 25) showed the same trend. The FAT1 expression increased in stage III and stage IV with a trend that approached statistical significance with P = 0.069 and P = 0.053. Likewise, we attempted AUC analysis at the protein level, also confirming a prominence in its diagnostic value (AUC = 0.79 in HNSC and AUC = 0.829 in OSCC) (**Figures 2B, C**). The protein expression level of FAT1 in 8 paired OSCC tissues was further verified *via* Western blotting (P < 0.001; **Figure 2D**).

**Table 2:** Expression levels of FAT1 in OSCC samples with different clinical and pathological characteristics.

Characteristics	Tumor center				Tumor front			
	Cases	FAT1+	FAT1-	P value	cases	FAT1+	FAT1-	P value
								
Tumor	118	65	53	<0.0001	107	67	40	<0.0001
Normal	116	3	113	****	116	3	113	****
**Gender**	117				106			
Female	37	20	17	0.8245	32	20	12	0.9209
Male	80	45	35		74	47	27	
**Age**	117				106			
Less than 55	53	35	18	0.0379	44	34	10	0.0114
55 and up	64	30	34	*	62	33	29	*
**Tumor stage**	116				105			
T1	16	7	9	0.7127	17	9	8	0.1758
T2	34	18	16		31	16	15	
T3	21	12	9		17	11	6	
T4	45	27	18		40	30	10	
**Differentiation**	117				106			
Poorly	11	9	2	0.1914	11	10	1	0.0203
Moderately	70	36	34		64	34	30	*
Highly	36	20	16		31	23	8	
**Metastasis**	117				106			
Yes	76	35	41	0.0063	71	40	31	0.0532
No	41	30	11	**	35	27	8	
**Depth of invasion**	100				90			
above median	50	30	20	0.4214	44	30	14	0.124
less than median	50	25	25		46	23	23	
**Extranodal extension**	96				86			
Negative	78	41	37	0.2106	71	42	29	0.8025
Positive	18	13	5		15	10	5	
**Perineural invasion**	102				92			
Negative	63	30	33	0.0535	57	30	27	0.1173
Positive	39	27	12		35	25	10	
**Tumor budding**	102				92			
Negative	78	38	40	0.0168	72	40	32	0.1898
Positive	24	19	5	*	20	15	5	

*P < 0.05; **P < 0.01; ****P < 0.0001.

### Analysis of FAT1 Mutations in Combined With TCGA HNSC and OSCC Tissues

**Figure 3:**
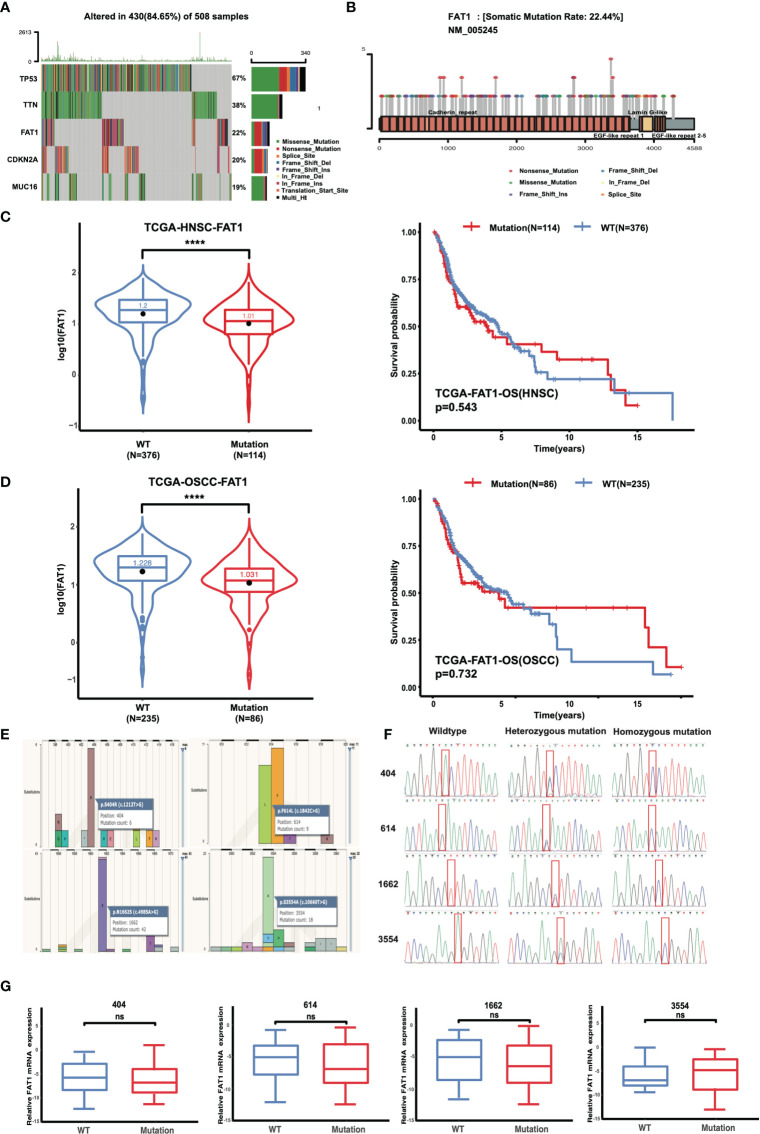
FAT1 exhibits high genetic alterations in HNSC and FAT1 mutation status and the affection of the FAT1 expression. **(A)** FAT1 was a highly mutated gene (22%) in HNSC. **(B)** Frequency of FAT1 mutation sites in HNSC. **(C)** FAT1 mRNA expression status between FAT1 wild-type and FAT1 mutation tumor tissues in HNSC from TCGA (boxplot and violin plot); Kaplan–Meier survival curve of OS time between in HNSC FAT1 wild-type and FAT1 mutation tumor tissues from TCGA. **(D)** FAT1 mRNA expression status between FAT1 wild-type and FAT1 mutation tumor tissues in OSCC from TCGA (boxplot and violin plot); Kaplan–Meier survival curve of OS time between in OSCC FAT1 wild-type and FAT1 mutation tumor tissues from TCGA. **(E)** Four chosen sites of the FAT1 from all types of cancers in COSMIC online. **(F)** Representative image of Sanger confirmation of four variant sites. **(G)** Relative FAT1 mRNA expression status between FAT1 wild-type and FAT1 variants tumor tissues in clinical samples according to four variants sites (boxplot). ns, P > 0.05; ****P < 0.0001.

Mutations of oncogenes or tumor-suppressor genes often result in deregulation of the expression of these genes themselves, such as TP53. For illustrative purposes, bioinformatics analysis of the FAT1 mutational profile in TCGA HNSC cohort (n = 508) were performed, and the results generated that the most mutated driver genes in HNSC included TP53, TTN, FAT1, CDKN2A, and MUC16 (**Figure 3A**). The 22% (114/508) alterations of FAT1 in HNSC were investigated, and most of those were missense or nonsense mutation, frameshift, or splice site, occurring mostly in the cadherin domains (**Figure 3B**). We evaluated the effect of altered FAT1 mutants and demonstrated that compared to the FAT1 wild-type group, the FAT1 mutant group conferred a significantly lower FAT1 expression level but not a specific survival correlation (P < 0.0001; **Figure 3C**). The results between wild-type samples (n = 235) and FAT1 mutant samples (n = 86) showed the same trend in OSCC (**Figure 3D**). Then, the next section of the survey was concerned with FAT1 mutation in OSCC tissues. Simultaneously, based on the COSMIC mutation database, no hotspot region was observed for FAT1 mutations in HNSC, so we selected 4 most frequent mutation sites (404, 614, 1,662, 3,554) detected in multiple cancers, which may have implications for protein function, to be validated by Sanger sequencing (**Figures 3E, F**). However, we inspected a high FAT1 mutation ratio in OSCC tissues but also in matched adjacent normal tissues and leukocyte DNA of normal individuals (**Table 3**). What is more, no significant association was detected between the candidate variants and FAT1 expression level in OSCC tissues (P > 0.05), but we could observe a decreasing FAT1 level trend in the FAT1 404, 614, and 1,662 mutated group (**Figure 3G**). Our results of mutation failed to identify the association between candidate sites and FAT1 expression level. They may be four SNP loci.

**Table 3:** FAT1 mutation status among OSCC tissues, matched adjacent normal tissues, and leukocyte DNA of normal individuals.

Sample	Case	FAT1-404			FAT1-614			FAT1-1662			FAT1-3554		
**UCSC**		ACT(-)			GAA(-)			GTT(-)			AGA(-)		
**Mutant**		CCT(-)			CAA(-)			GCT(-)			AGC(-)		
**Mutant(aa)**		S404R			F614L			N1662S			S3554A		
		A/A	A/C	C/C	G/G	G/C	C/C	T/T	T/C	C/C	A/A	A/C	C/C
**Tumor**	58	32	24	2	10	32	16	18	29	11	10	22	25
**Adjacent normal**	58	31	25	2	10	32	16	18	31	8	10	23	25
**Healthy control**	26	17	7	2	7	14	5	5	16	5	2	9	16

### Downregulation of FAT1 Suppresses Proliferation, Stemness and Cell Cycle, and Promoted Cell Apoptosis in OSCC Cells

**Figure 4:**
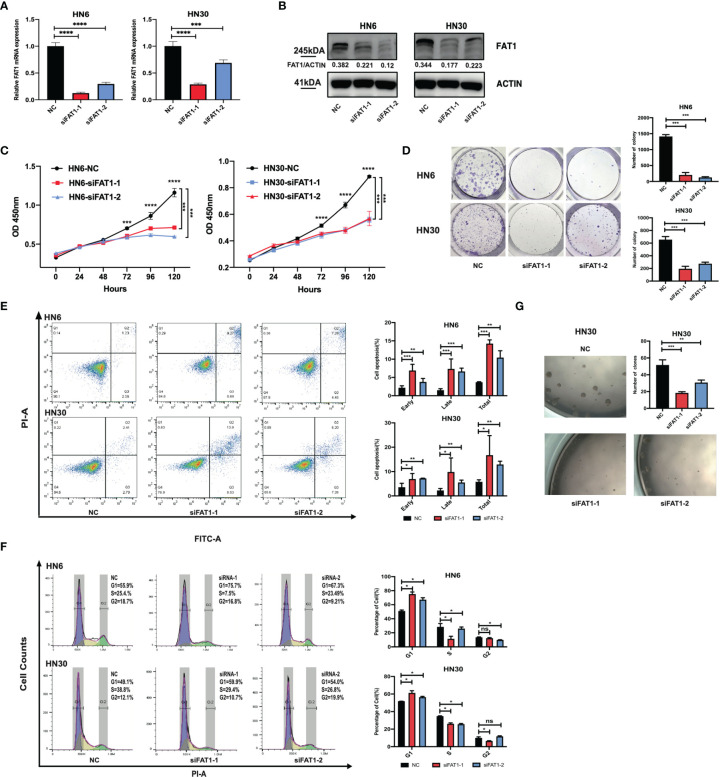
Downregulation of FAT1 impairs OSCC proliferation, cell cycle, and stemness, and promoted cell apoptosis. FAT1 knockdown by siFAT1-1 and siFAT1-2 in both HN6 and HN30 was confirmed by qPCR **(A)** and Western blot **(B)**. FAT1 knockdown by siFAT1-1 and siFAT1-2 inhibited HN6 and HN30 cell proliferation *via* CCK8 analysis **(C)** and clone formation **(D)**, facilitated cell both early and late apoptosis **(E)**, promoted cell cycle arrest **(F)**, and suppressed the tumorsphere formation of HN30 **(G)**. *P < 0.05; **P < 0.01; ***P < 0.001; ****P < 0.0001; ns, not significant.

To explore potential FAT1 functions in OSCC, we conducted a loss-of-function study using two small interfering RNAs to knock down FAT1 in the HN6 and HN30 cell lines *in vitro*. Compared with the negative control siRNA group, RT-qPCR and Western blots determined the downregulated FAT1 expression in both mRNA and protein levels in the two-siRNA group (siFAT1-1 and siFAT1-2) (**Figures 4A, B**). First, we applied CCK-8 and colony formation assays to evaluate the functional effects of FAT1 alterations on cell growth. The results revealed that cell proliferation was suppressed after FAT1 silencing in both cell lines (**Figures 4C, D**). Meanwhile, the downregulation of FAT1 promoted OSCC cell apoptosis, including early apoptosis and late apoptosis (**Figure 4E**), and an increase in proportion in the G0/G1 phase accompanied by a decrease in proportion in the S phase was observed, hindering G1/S progression (**Figure 4F**). Due to the observation of the trend of significant inhibition of growth, we hypothesize that FAT1 expression contributes to the maintenance of OSCC CSCs. With tumorsphere assay, the self-renewal capability of HN30 was found to be suppressed after the knockdown of FAT1 (**Figure 4G**) while HN6 itself lacked the sphere-forming ability. Taken together, these results demonstrated that FAT1 may act as a tumor suppressor in OSCC.

### FAT1 Increases Cell Migration *In Vitro*

**Figure 5:**
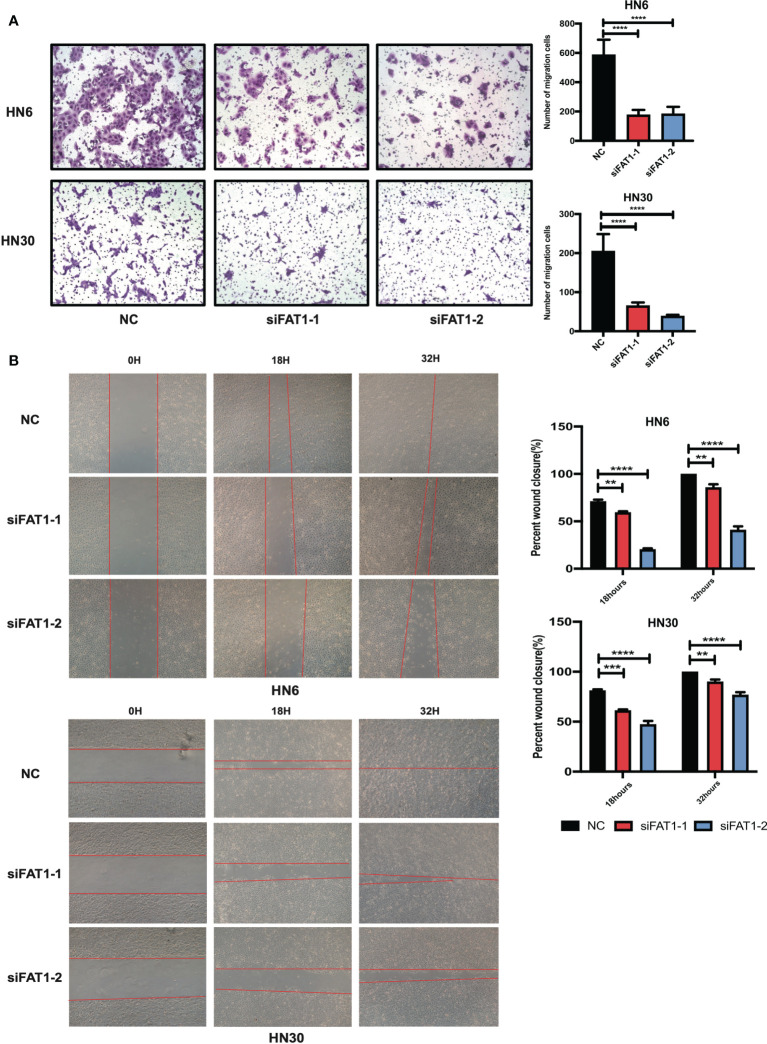
Downregulation of FAT1 impairs migration ability of OSCC. Knockdown by siFAT1-1 and siFAT1-2 inhibited HN6 and HN30 cells migration *via* transwell chambers without Matrigel **(A)** and wound healing migration assays **(B)**. **P < 0.01; ***P < 0.001; ****P < 0.0001.

We next examined whether FAT1 could impact the migration of OSCC cells. Taking advantage of the transwell assay, the results showed that FAT1 knockdown decreased the migrated cell numbers (**Figure 5A**). The results of the wound-healing assay displayed that, 18 h after scratching, the blank areas in the negative control were wider than in the interfering group and after 32 h, the negative control cells almost healed, while there were wide areas in the interfering group (**Figure 5B**). Thus, we validated that knockdown of FAT1 prevented OSCC cell migration.

### RNA Sequencing Indicates That FAT1 Plays an Important Role in Cell Proliferation and Activated Pathways

**Figure 6:**
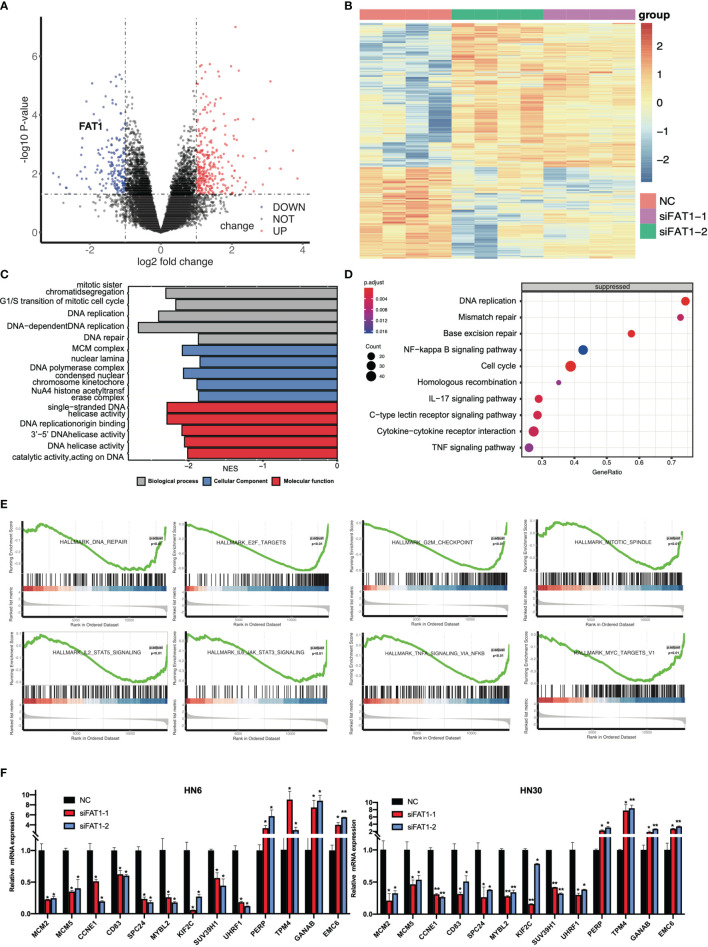
Results of the RNA sequencing analysis and validation of DEGs. **(A)** Volcano plots of the data from RNA-seq analysis by the Limma-voom R package, presenting the differences between NC and FAT1-KD groups and plotting the log2(fold change) versus the –log10(adjusted P-value). 472 differentially expressed genes (DEGs) were confirmed, comprising 289 upregulated (red dots) and 183 downregulated (blue dots) using the thresholds P < 0.05 and abs (log2 fold change)> 1. **(B)** Heat map of the DEGs. **(C)** Suppressed enriched GO terms (BP, CC, and MF). **(D)** Suppressed enriched KEGG terms. **(E)** Gene set enrichment analysis (GSEA) plots of the major enriched hallmark pathway. **(F)** Validation of DEGs of RNA-seq results through quantitative PCR. *P < 0.05; **P < 0.01.

**Figure 7:**
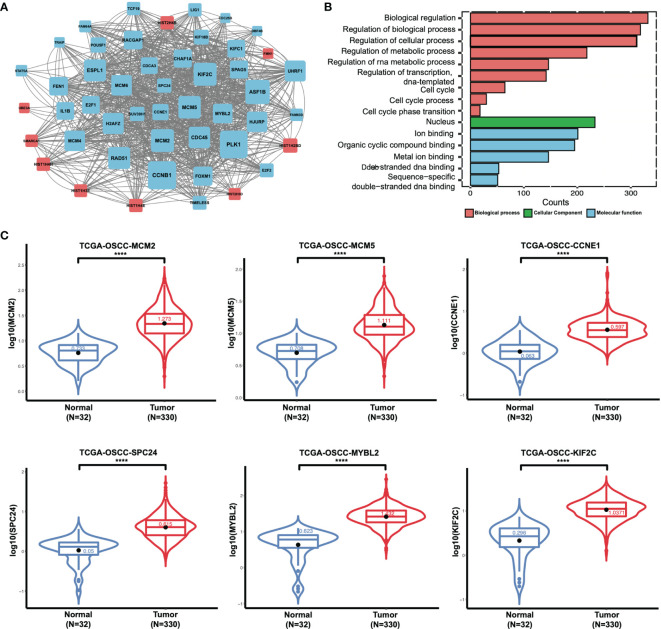
Results of the RNA sequencing analysis and validation of DEGs. **(A)** Protein–protein interactive network of DEGs with nodes >19 and their related proteins. The different sizes of the nodes represented the degree of the node specific in the PPI network. Blue nodes mean downregulation level while red nodes represent upregulation level. **(B)** Enriched GO terms of DEGs (BP, CC, and MF). **(C)** MCM2, MCM5, CCNE1 SPC24, MYBL2, and KIF2C mRNA expression status between normal tissues and tumor tissues in OSCC from TCGA (boxplot and violin plot). ****P < 0.0001.

To delineate transcriptomic changes after suppression of FAT1 in OSCC oncogenesis, RNA sequencing was performed after FAT1 knockdown (siFAT1-1 and siFAT1-2) compared to the control group (NC) in two OSCC cells; the raw data have been deposited in the NCBI gene expression omnibus (GEO) under accession no. GSE196138 (https://www.ncbi.nlm.nih.gov/geo/query/acc.cgi?acc=GSE196138). After bioinformatic analysis, volcano plots were used to assess the variation in gene expression (**Figure 6A**). Totally, 472 differentially expressed genes were confirmed, comprising 289 upregulated and 183 downregulated using the thresholds P < 0.05 and abs (log2 fold change)> 1 *via* Limma-voom methods. The heat map consisted of DEGs revealed tight clustering, separating the FAT1 KD group and the normal control group in both two cell lines into two distinct clusters (**Figure 6B**). We then performed gene-set enrichment analysis in an order of decreasing log2 fold change as a ranked gene list of all genes. Consistent with our observations *in vitro*, GO analysis reflected that the affected genes by FAT1 knockdown were involved most significantly in cell death, survival, and proliferation. We showed 5 most significantly enriched terms (according to q values or NES) from the cellular component (CC), biological process (BP), and molecular function (MF; **Figure 6C**). Kyoto Encyclopedia of Genes and Genomes analysis revealed that in addition to the signaling pathways associated with cell growth similar to the above, FAT1 KD suppressed the TNF, NF-kappa B, and IL-17 signaling pathway (**Figure 6D**). Moreover, we conducted a GSEA analysis using the hallmark gene set from the Molecular Signatures Database (mSigDB, http://software.broadinstitute.org/gsea/index.jsp) and observed that E2F targets, G2M checkpoint, TNFA signaling up NFKB, DNA repair, mitotic spindle, IL6 JAK STAT3 signaling, IL2 STAT5 signaling, and MYC targets V1 were negatively correlated with FAT1 expression (**Figure 6E**), corroborating our above findings. To validate the RNA-seq data, 13 DEGs, including 8 genes (MCM2, MCM5, CCNE1, CD83, SPC24, MYBL2, KIF2C, SUV39H1) involved in E2F targets, G2M checkpoint, TNFA signaling up NFKB, IL2 STAT5 signaling, and MYC targets V1 as core enrichment and other 5 randomly selected genes (UHRF1, PERP, TPM4, GANAB, EMC6), were determined *via* qRT-PCR analysis. The change trends were consistent with those detected *via* transcriptome sequencing (**Figure 6F**). PPI network constriction was serviced to visualize DEG protein–protein interactions. The different sizes of the nodes represented the degree of the node in the PPI network. In the network, we visualized them with different sizes of the nodes and the label represents the degree of the node of the top 30 genes which contain at least 19 nodes with related interaction genes. Blue nodes mean downregulation level while red nodes represent upregulation level (**Figure 7A**). Similarly, the enriched terms in the Gene Ontology were mainly related to the cell cycle and replication (**Figure 7B**). We focused on six hub DEGs with more than 19 nodes also involved in the pathway signaling mentioned above and determined *via* qRT-PCR analysis. The expression of MCM2, MCM5, CCNE1 SPC24, MYBL2, and KIF2C was significantly upregulated in OSCC samples (**Figure 7C**). These results implicated FAT1 as a factor mediating the growth characteristics of OSCC cells.

## Discussion

Oral squamous cell carcinoma is a major subset of head and neck squamous cell carcinoma, which is one of the most common malignant tumors globally with a high rate of genetic heterogeneity, resulting in loss-of-function mutations in tumor-suppressor genes and activation of oncogenes (29, 30). The studies from Morris et al. showed that the recurrent somatic mutation of FAT1 was detected in several cancer types and FAT1 mutation could result in promotion of WNT signaling in GBM pointing FAT1 to act as a tumor suppressor. There were 6 heterozygous mutations in head and neck squamous cell carcinoma (4 of 60; 6.7%), but they did not evaluate the function in OSCC cells (8). Nakaya et al. reported that FAT1 was homozygously deleted in 80% of primary oral cancer cases and FAT1 mRNA expression was repressed because of homozygous deletion and/or promoter CpG hypermethylation (31, 32). Non-synonymous FAT1 mutations have been linked to better prognosis in HPV-negative HNSC patients (33). Lin et al. reported that FAT1 mRNA and protein expression was downregulated or absent compared with that in normal oral mucosal epithelial cells in HNSC cell lines (SCC25, OECM1, FaDu, HSC3, SAS, and OC3) (18). However, research results from Hsu et al. demonstrated that FAT1 could promote the process of the tumor (22). Hence, in the present research, we first checked the expression levels of FAT1 comprehensively. Both the data from clinical specimens and the data from the public database (TCGA, GEO, and CPTAC) indicated that the expression of FAT1 was upregulated in HNSC and OSCC in both mRNA and protein levels and a high FAT1 expression level correlated with poor prognosis. In the IHC assay, we observed an upregulation of FAT1 in tumor tissues, but the survival difference did not reach statistical significance. The positive FAT1 staining which resulted for these parameters was associated with age, differentiation, metastasis, and tumor budding, indicating that FAT1 has potential for clinical pathologic diagnosis. Through AUC, both FAT1 mRNA and protein levels performed well in distinguishing tumor and normal tissues (AUC = 0.78). These results indicate that FAT1 is closely related with the neoplasia and development of HNSC and OSCC.

To advance the understanding of HNSC, the CPTAC program, which has been publicly available in 2020, has performed a comprehensive integrated proteogenomic characterization of a 109 human papilloma virus (HPV)-negative HNSC cohort (34). FAT1 is one of the most frequently mutated genes in this cohort, in which truncating mutations account for >70% of all FAT1 mutations, in sharp contrast to other cancer types. However, none of the previous studies, which have linked FAT1 mutations to the WNT and HIPPO pathways, were supported by this cohort (8, 19). Instead, an investigation of the mutually exclusive relationship between FAT1 truncating mutations and 11q13.3 amplifications revealed their functional convergence on dysregulated actin dynamics, underlying poor prognosis of tumors with these genetic aberrations (35). We also explored FAT1 protein expression levels in this cohort. FAT1 was expressed remarkably high in tumor tissues compared to normal tissues and in stage IV compared to stage I tumor tissues in the whole cohort (P < 0.01). Focusing on OSCCs (53/109), FAT1 protein expression was upregulated remarkably and increased in stage III and stage IV with a trend that approached statistical significance with P = 0.069 and P = 0.053. The FAT1 protein level also performed well in distinguishing tumor and normal tissues in HNSC and OSCC (AUC = 0.79 and 0.829).

Lin et al. performed multiplex polymerase chain reaction-based next-generation sequencing to indicate that approximately 29% of HNSC patients harbored damaging non-synonymous FAT1 mutations, which could be prognostic indicators (18). Martin et al. conducted a pan-cancer analysis and recognized only FAT1 to be significantly mutated (19). Moreover, in a recent newly genomic analysis, FAT1 has also been identified (an early-onset OTSCC cohort, which was diagnosed before the age of 50 years, and a novel buccal mucosal cancer cell line “GBC035” derived from non-tobacco users) (36, 37). We explored FAT1 mutation status in TCGA HNSC and OSCC and demonstrated that FAT1 mutation did correlate with reduced mRNA levels but not survival outcomes in both HPV+ and HPV- patients. For 404, 614, 1,662, and 3,554, the four FAT1 hot mutation sites in all types of cancers in the COSMIC database, we observed a high mutation ratio in clinical OSCC tissues but also in matched adjacent normal tissues and leukocyte DNA of normal individuals.

Next, knocking down the FAT1 expression showed that downregulated FAT1 could inhibit tumor formation and progression by inhibiting cell proliferation, stemness, and cell cycle and promoting the apoptosis. Furthermore, the ability of migration was suppressed by FAT1 silencing. The analysis of RNA-seq constructed by FAT1 knockdown (siFAT1-1 and siFAT1-2) compared to the control group supported FAT1 as a factor mediating the growth characteristics of OSCC cells through DNA repair, cell cycle, DNA repair, and other signaling pathways, which were pivotal mechanisms influencing cell proliferation, cycle, apoptosis, and migration. GSEA analyses reflect processes or pathways affected by FAT1 downregulation, e.g., those related to cellular proliferation (E2F targets, G2M checkpoint, TNFA signaling up NFKB, mitotic spindle, IL6 JAK STAT3 signaling, IL2 STAT5 signaling, and MYC targets) and the DNA damage responses. We further focused on six of the most important DEGs (MCM2, MCM5, CCNE1 SPC24, MYBL2, KIF2C), which were involved in the above pathway that may exert a regulatory function in OSCC with a significant upregulation expression level in TCGA as well as the hub genes in the PPI network with DEGs. Their downregulation with FAT1 silencing was proved by qPCR. As a result, additional focus on the molecular mechanism of the six hub genes in OSCC is warranted. However, it still lacked experimental exploration of assumed FAT1-related genes to validate our speculations. The study of Lin et al. disagreed with ours, and we carefully attributed this to the genetic heterogeneity that reflected the complicated constitution of different HNSC cells (18). Otherwise, our findings of FAT1, partly corroborated by data of HSC-3 and SAS of Hsu et al., showed two human squamous carcinomas of the tongue cells which exhibited high lymph node metastasis potential and poor differentiation status (38). Hsu et al. found that FAT1 played a role in the regulation of oral carcinogenesis and cisplatin resistance through deregulation of the LRP5/WNT2/GSS signaling axis (22). Therefore, our current study provided a reason for FAT1 to jump out of its classic role as a tumor suppressor by featuring its oncogenic properties mainly *via* cellular proliferation and repair.

## Conclusions

In summary, we illuminated that FAT1 was upregulated in OSCC and correlated with a poor survival and disease progression. No relationship was observed between the variants of these loci (404, 614, 1,662, and 3,554) and tumorigenesis or FAT1 expression. We found that FAT1 could promote OSCC cell proliferation, cell cycle, and migration and inhibit cell apoptosis in an *in vitro* experiment. RNA-seq analysis of FAT1 silencing revealed that the cell cycle, DNA replication, and some core genes (MCM2, MCM5, CCNE1 SPC24, MYBL2, KIF2C) may be the potential mechanisms in OSCC, which was consistent with the *in vitro* phenotype experiments. Therefore, these findings provided new insight into the role of FAT1 as an oncogene and its more potential mechanisms.

## Data Availability Statement

The datasets presented in this study can be found in online repositories. The names of the repository/repositories and accession number(s) can be found as follows: https://www.ncbi.nlm.nih.gov/geo/query/acc.cgi?acc=GSE196138.

## Ethics Statement

The studies involving human participants were reviewed and approved by Fujian Medical University School and Hospital of Stomatology. The patients/participants provided their written informed consent to participate in this study.

## Author Contributions

DZ and YL presented the study concepts. TL, DZ, and YL designed this study. TL, QG, KZ, LH, and YY were in charge of the data acquisition. TL, QG, and KZ controlled the quality of data and algorithms. TL, QG, and LZ analyzed and interpreted the data. TL, LZ, and DZ performed the statistical analysis. TL, LZ, and DZ wrote the first draft of the manuscript. All authors contributed to the manuscript revision and read and approved the submitted version.

## Funding

This work was supported by the National Natural Sciences Foundation of China (grant number 81872186), grants from Joint Funds for the Innovation of Science and Technology, Fujian province (grant number: 2019Y9112), Natural Sciences Foundation of Fujian Province (grant number: 2019J01318), and scientific research funding of the School and Hospital of Stomatology, Fujian Medical University (grant number: 2018KQYJ01). The funding bodies had no role in the design of the study and collection, analysis, and interpretation of data and in writing of the manuscript.

## Conflict of Interest

The authors declare that the research was conducted in the absence of any commercial or financial relationships that could be construed as a potential conflict of interest.

## Publisher’s Note

All claims expressed in this article are solely those of the authors and do not necessarily represent those of their affiliated organizations, or those of the publisher, the editors and the reviewers. Any product that may be evaluated in this article, or claim that may be made by its manufacturer, is not guaranteed or endorsed by the publisher.

## References

[1] Adelstein D Gillison ML Pfister DG Spencer S Adkins D Brizel DM. NCCN Guidelines Insights: Head and Neck Cancers, Version 2. 2017. J Natl Compr Cancer Netw (2017) 15(6):761–70. doi: 10.6004/jnccn.2017.0101 28596256

[2] Leemans CR Braakhuis BJM Brakenhoff RH. The Molecular Biology of Head and Neck Cancer. Nat Rev Cancer (2010) 11(1):9–22. doi: 10.1038/nrc2982 21160525

[3] Campbell KM Lin T Zolkind P Barnell EK Skidmore ZL Winkler AE. Oral Cavity Squamous Cell Carcinoma Xenografts Retain Complex Genotypes and Intertumor Molecular Heterogeneity. Cell Rep (2018) 24(8):2167–78. doi: 10.1016/j.celrep.2018.07.058 PMC6417872 30134176

[4] Kalu NN Mazumdar T Peng S Shen L Sambandam V Rao X. Genomic Characterization of Human Papillomavirus-Positive and -Negative Human Squamous Cell Cancer Cell Lines. Oncotarget (2017) 8(49):86369–83. doi: 10.18632/oncotarget.21174 PMC5689691 29156801

[5] Sadeqzadeh E de Bock CE Thorne RF. Sleeping Giants: Emerging Roles for the Fat Cadherins in Health and Disease. Medicinal Res Rev (2013) 34(1):190–221. doi: 10.1002/med.21286 23720094

[6] Dunne J Hanby AM Poulsom R Jones TA Sheer D Chin WG. Molecular Cloning and Tissue Expression of FAT, the Human Homologue of the Drosophila Fat Gene That Is Located on Chromosome 4q34–Q35 and Encodes a Putative Adhesion Molecule. Genomics (1995) 30(2):207–23. doi: 10.1006/geno.1995.9884 8586420

[7] Yaoita E Kurihara H Yoshida Y Inoue T Matsuki A Sakai T. Role of Fat1 in Cell-Cell Contact Formation of Podocytes in Puromycin Aminonucleoside Nephrosis and Neonatal Kidney. Kidney Int (2005) 68(2):542–51. doi: 10.1111/j.1523-1755.2005.00432.x 16014031

[8] Morris LGT Kaufman AM Gong Y Ramaswami D Walsh LA Turcan Ş. Recurrent Somatic Mutation of FAT1 in Multiple Human Cancers Leads to Aberrant Wnt Activation. Nat Genet (2013) 45(3):253–61. doi: 10.1038/ng.2538 PMC3729040 23354438

[9] Li Z Razavi P Li Q Toy W Liu B Ping C. Loss of the FAT1 Tumor Suppressor Promotes Resistance to CDK4/6 Inhibitors *via* the Hippo Pathway. Cancer Cell (2018) 34(6):893–905.e8. doi: 10.1016/j.ccell.2018.11.006 30537512 PMC6294301

[10] Wang L Lyu S Wang S Shen H Niu F Liu X. Loss of FAT1 During the Progression From DCIS to IDC and Predict Poor Clinical Outcome in Breast Cancer. Exp Mol Pathol (2016) 100(1):177–83. doi: 10.1016/j.yexmp.2015.12.012 26721716

[11] Wang Y Wang G Ma Y Teng J Wang Y Cui Y. FAT1, a Direct Transcriptional Target of E2F1, Suppresses Cell Proliferation, Migration and Invasion in Esophageal Squamous Cell Carcinoma. Chin J Cancer Res (2019) 31(4):609–19. doi: 10.21147/j.issn.1000-9604.2019.04.05 PMC6736659 31564804

[12] Lu Y Wang Z Zhou L Ma Z Zhang J Wu Y. FAT1 and PTPN14 Regulate the Malignant Progression and Chemotherapy Resistance of Esophageal Cancer Through the Hippo Signaling Pathway. Anal Cell Pathol (2021) 2021:1–9. doi: 10.1155/2021/9290372 PMC8548181 34712552

[13] Yu J Li H. The Expression of FAT1 Is Associated With Overall Survival in Children With Medulloblastoma. Tumori J (2016) 103(1):44–52. doi: 10.5301/tj.5000570 27834469

[14] De Bock CE Ardjmand A Molloy TJ Bone SM Johnstone D Campbell DM. The Fat1 Cadherin Is Overexpressed and an Independent Prognostic Factor for Survival in Paired Diagnosis–Relapse Samples of Precursor B-Cell Acute Lymphoblastic Leukemia. Leukemia (2011) 26(5):918–26. doi: 10.1038/leu.2011.319 22116550

[15] Neumann M Seehawer M Schlee C Vosberg S Heesch S von der Heide EK. FAT1 Expression and Mutations in Adult Acute Lymphoblastic Leukemia. Blood Cancer J (2014) 4(6):e224. doi: 10.1038/bcj.2014.44 24972153 PMC4080215

[16] Valletta D Czech B Spruss T Ikenberg K Wild P Hartmann A. Regulation and Function of the Atypical Cadherin FAT1 in Hepatocellular Carcinoma. Carcinogenesis (2014) 35(6):1407–15. doi: 10.1093/carcin/bgu054 24590895

[17] Meng P Zhang Y-F Zhang W Chen X Xu T Hu S. Identification of the Atypical Cadherin FAT1 as a Novel Glypican-3 Interacting Protein in Liver Cancer Cells. Sci Rep (2021) 11(1):40. doi: 10.1038/s41598-020-79524-3 33420124 PMC7794441

[18] Lin S-C Lin L-H Yu S-Y Kao S-Y Chang K-W Cheng H-W. FAT1 Somatic Mutations in Head and Neck Carcinoma Are Associated With Tumor Progression and Survival. Carcinogenesis (2018) 39(11):1320–30. doi: 10.1093/carcin/bgy107 30102337

[19] Martin D Degese MS Vitale-Cross L Iglesias-Bartolome R Valera JLC Wang Z. Assembly and Activation of the Hippo Signalome by FAT1 Tumor Suppressor. Nat Commun (2018) 9(1):2372. doi: 10.1038/s41467-018-04590-1 29985391 PMC6037762

[20] Dikshit B Irshad K Madan E Aggarwal N Sarkar C Chandra PS. FAT1 Acts as an Upstream Regulator of Oncogenic and Inflammatory Pathways, *via* PDCD4, in Glioma Cells. Oncogene (2012) 32(33):3798–808. doi: 10.1038/onc.2012.393 22986533

[21] Madan E Dikshit B Gowda SH Srivastava C Sarkar C Chattopadhyay P. FAT1 Is a Novel Upstream Regulator of HIF1α and Invasion of High Grade Glioma. Int J Cancer (2016) 139(11):2570–82. doi: 10.1002/ijc.30386 PMC6585695 27536856

[22] Hsu TN Huang CM Huang CS Huang MS Yeh CT Chao TY. Targeting FAT1 Inhibits Carcinogenesis, Induces Oxidative Stress and Enhances Cisplatin Sensitivity Through Deregulation of LRP5/WNT2/GSS Signaling Axis in Oral Squamous Cell Carcinoma. Cancers (2019) 11(12):1883. doi: 10.3390/cancers11121883 PMC6966489 31783581

[23] Colaprico A Silva TC Olsen C Garofano L Cava C Garolini D. TCGAbiolinks: An R/Bioconductor Package for Integrative Analysis of TCGA Data. Nucleic Acids Res (2015) 44(8):e71. doi: 10.1093/nar/gkv1507 26704973 PMC4856967

[24] Anand M De-Chen L Yassen A Christoph P Phillip KH. Maftools: Efficient and Comprehensive Analysis of Somatic Variants in Cancer. Genome Res (2018) 28(11):1747–56. doi: 10.1101/gr.239244.118 PMC6211645 30341162

[25] Zheng K Lan T Li G Huang L Chen Y Su BH. Evaluated Expression of CELSR3 in Oral Squamous Cell Carcinoma Is Associated With Perineural Invasion and Poor Prognosis. Oral Surg Oral Med Oral Pathol Oral Radiol (2021) S2212-4403(21)00714-8. doi: 10.1016/j.oooo.2021.10.016 35165064

[26] Cappuzzo F Hirsch FR Rossi E Bartolini S Ceresoli GL Bemis L. Epidermal Growth Factor Receptor Gene and Protein and Gefitinib Sensitivity in Non–Small-Cell Lung Cancer. J Natl Cancer Institute (2005) 97(9):643–55. doi: 10.1093/jnci/dji112 15870435

[27] Ritchie ME Phipson B Wu D Hu Y Law CW Shi W. Limma Powers Differential Expression Analyses for RNA-Sequencing and Microarray Studies. Nucleic Acids Res (2015) 43(7):e47. doi: 10.1093/nar/gkv007 25605792 PMC4402510

[28] Wu T Hu E Xu S Chen M Guo P Dai Z. ClusterProfiler 4.0: A Universal Enrichment Tool for Interpreting Omics Data. Innovation (2021) 2(3):100141. doi: 10.1016/j.xinn.2021.100141 34557778 PMC8454663

[29] Kemmer JD Johnson DE Grandis JR. Leveraging Genomics for Head and Neck Cancer Treatment. J Dental Res (2018) 97(6):603–13. doi: 10.1177/0022034518756352 PMC5960879 29420101

[30] Alsahafi E Begg K Amelio I Raulf N Lucarelli P Sauter T. Clinical Update on Head and Neck Cancer: Molecular Biology and Ongoing Challenges. Cell Death Dis (2019) 10(8):540. doi: 10.1038/s41419-019-1769-9 31308358 PMC6629629

[31] Katoh M. Function and Cancer Genomics of FAT Family Genes. Int J Oncol (2012) 41(6):1913–8. doi: 10.3892/ijo.2012.1669 PMC3583642 23076869

[32] Nakaya K Yamagata HD Arita N Nakashiro K Nose M Miki T. Identification of Homozygous Deletions of Tumor Suppressor Gene FAT in Oral Cancer Using CGH-Array. Oncogene (2007) 26(36):5300–8. doi: 10.1038/sj.onc.1210330 17325662

[33] Kim KT Kim B-S Kim JH. Association Between FAT1 Mutation and Overall Survival in Patients With Human Papillomavirus-Negative Head and Neck Squamous Cell Carcinoma. Head Neck (2016) 38(S1):E2021–9. doi: 10.1002/hed.24372 PMC5067633 26876381

[34] Mertins P Tang LC Krug K Clark DJ Gritsenko MA Chen L. Reproducible Workflow for Multiplexed Deep-Scale Proteome and Phosphoproteome Analysis of Tumor Tissues by Liquid Chromatography–Mass Spectrometry. Nat Protoc (2018) 13(7):1632–61. doi: 10.1038/s41596-018-0006-9 PMC6211289 29988108

[35] Huang C Chen L Savage SR Eguez RV Dou Y Li Y. Proteogenomic Insights Into the Biology and Treatment of HPV-Negative Head and Neck Squamous Cell Carcinoma. Cancer Cell (2021) 39(3):361–9. e16. doi: 10.1016/j.ccell.2020.12.007 33417831 PMC7946781

[36] Campbell BR Chen Z Faden DL Agrawal N Li RJ Hanna GJ. The Mutational Landscape of Early- and Typical-Onset Oral Tongue Squamous Cell Carcinoma. Cancer (2020) 127(4):544–53. doi: 10.1002/cncr.33309 PMC7891879 33146897

[37] Vipparthi K Patel AK Ghosh S Das S Das C Das K. Two Novel Cell Culture Models of Buccal Mucosal Oral Cancer From Patients With No Risk-Habits of Tobacco Smoking or Chewing. Oral Oncol (2021) 113:105131. doi: 10.1016/j.oraloncology.2020.105131 33387705

[38] Matsui T Ota T Ueda Y Tanino M Odashima S. Isolation of a Highly Metastatic Cell Line to Lymph Node in Human Oral Squamous Cell Carcinoma by Orthotopic Implantation in Nude Mice. Oral Oncol (1998) 34(4):253–6. doi: 10.1016/S1368-8375(98)80003-1 9813718

