# Supplementary material for: FAT1 Upregulates in Oral Squamous Cell Carcinoma and Promotes Cell Proliferation via Cell Cycle and DNA Repair
Source: Front Oncol. 2022 May 11;12:870055. doi: 10.3389/fonc.2022.870055 (PMC9130556; doi:10.3389/fonc.2022.870055)

Supplementary Data

All the raw data in this manuscript were listed in this file and another excel file. And the uncropped scanned membranes, microscopy images, flow cytometry data, the relevant code/script files and data sheets were deposited in Jianguoyun, which can be found online at:

https://www.jianguoyun.com/p/Dcy27SEQkr-hChjm9qsE

The raw data of RNA-sequencing have been deposited in the NCBI gene expression omnibus (GEO) under accession no. GSE196138 (https://www.ncbi.nlm.nih.gov/geo/query/acc.cgi?acc=GSE196138).

Fig2C

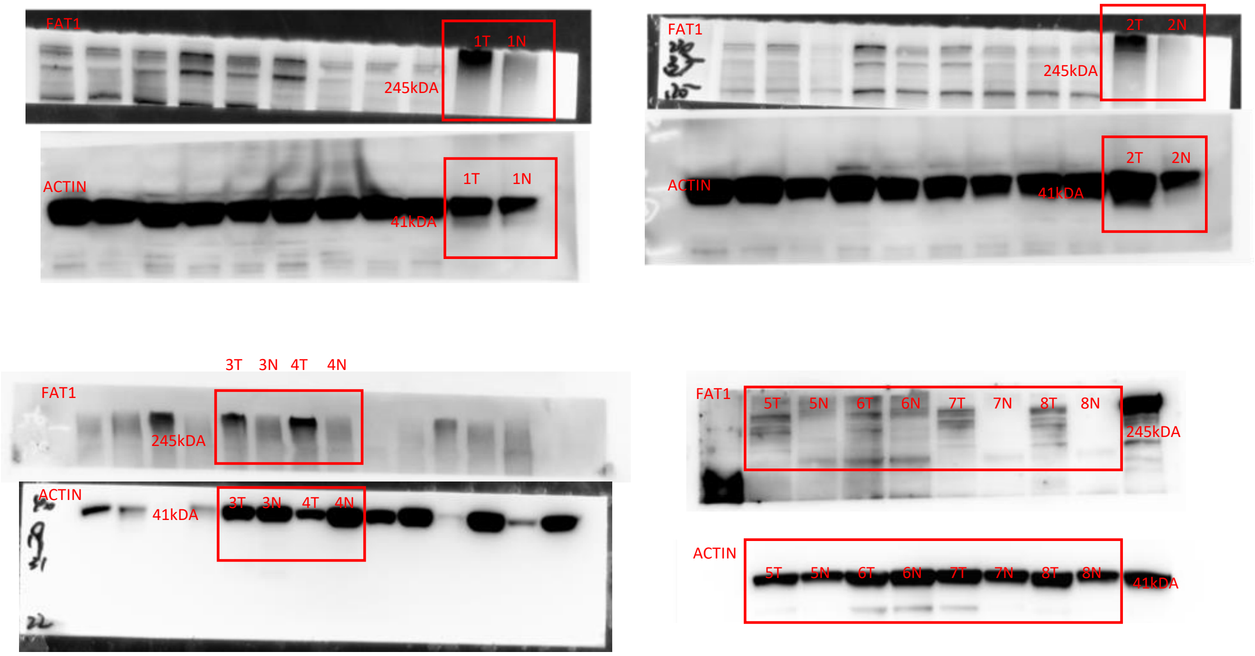

sample	1T	1N	2T	2N	3T	3N	4T	4N
FAT1	123647	21186	113858	17597	76794	32550	79497	15799
ACTIN	311236	253617	356137	253421	191457	197982	136623	226158
FAT1/ACTIN	0.3972	0.0835	0.3197	0.0693	0.4011	0.1687	0.5818	0.0698
sample	5T	5N	6T	6N	7T	7N	8T	8N
FAT1	47470	11267	44208	18420	44589	1053	52166	1294
ACTIN	127272	100016	140860	150689	138132	120342	148991	12065
FAT1/ACTIN	0.3729	0.1126	0.3134	0.0122	0.3264	0.0087	0.3501	0.0107

Points with image J software

Fig4B

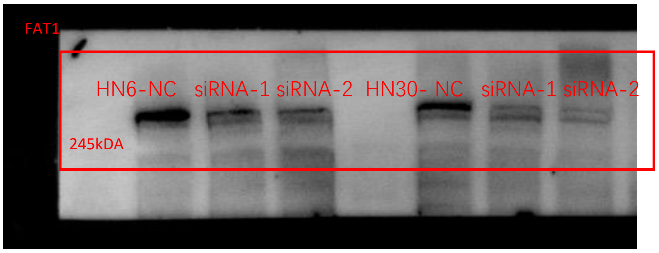

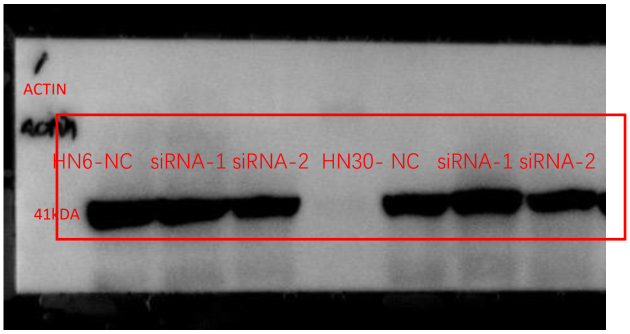

Points with image J software

Sample	HN6-NC	HN6-siRNA-1	HN6-siRNA-2	HN30-NC	HN30-siRNA-1	HN30-siRNA-2
FAT1	129334	64087	37645	87522	42302	26067
ACTIN	196137	208546	158328	162327	181471	158251
FAT1/ACTIN	0.6591	0.3073	0.2377	0.5329	0.2696	0.1646

Western blot of another independent repeat of silencing FAT1 by siRNA

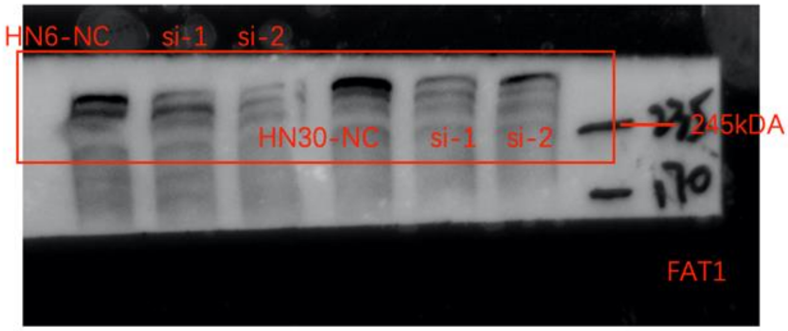

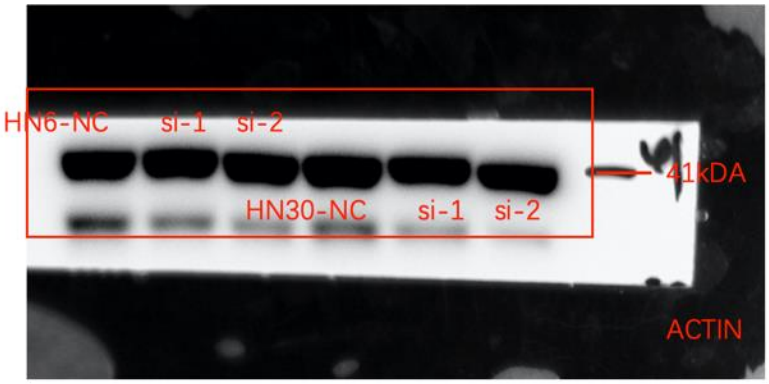

Points with image J software

Sample	HN6-NC	HN6-siRNA-1	HN6-siRNA-2	HN30-NC	HN30-siRNA-1	HN30-siRNA-2
FAT1	40310	23536	13154	41497	20355	25310
ACTIN	105523	106735	110388	120596	114893	113293
FAT1/ACTIN	0.382	0.221	0.120	0.344	0.177	0.223

Fig4D

Number of clones

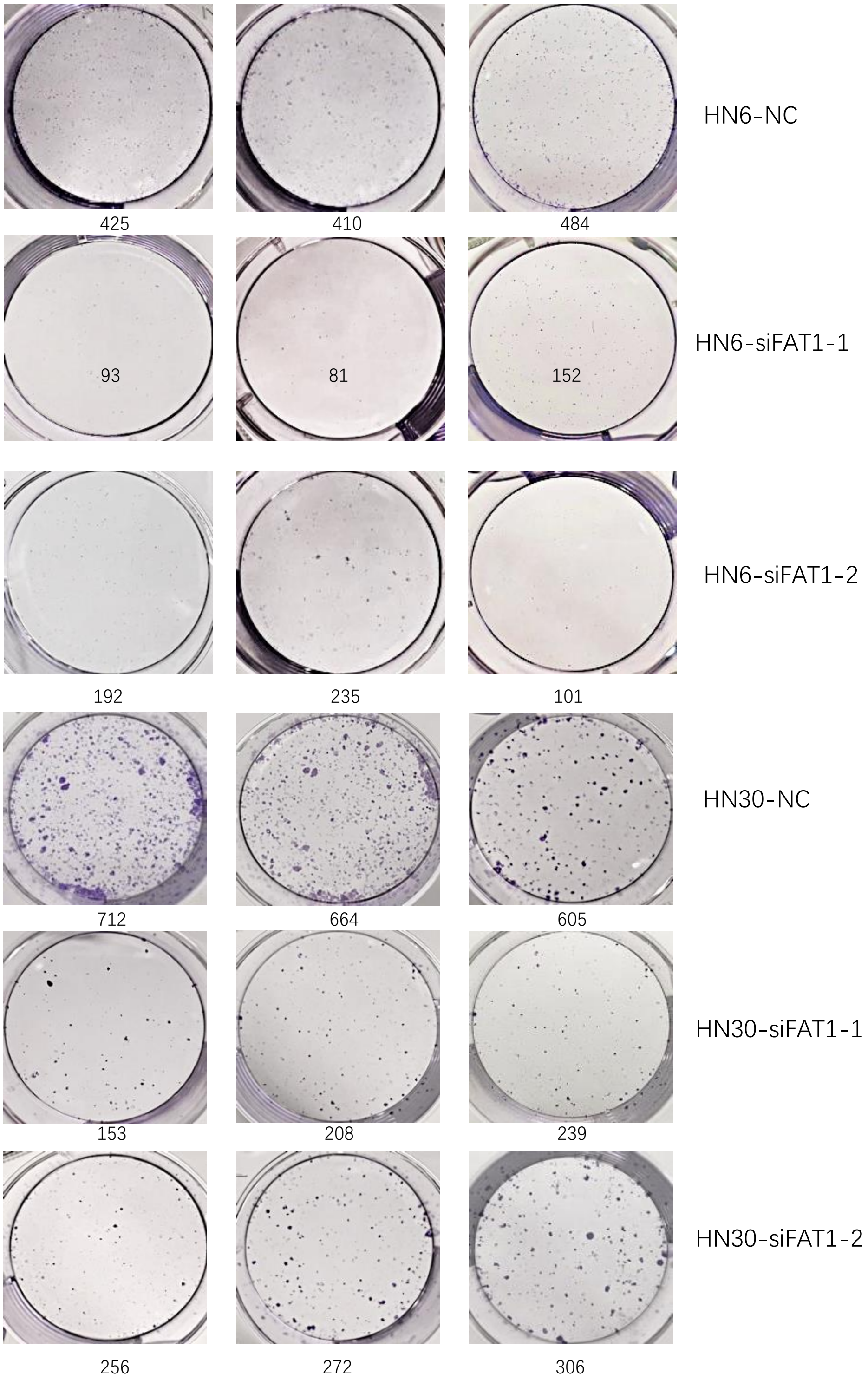

Number of clones of another independent repeat clone formation assay of HN6

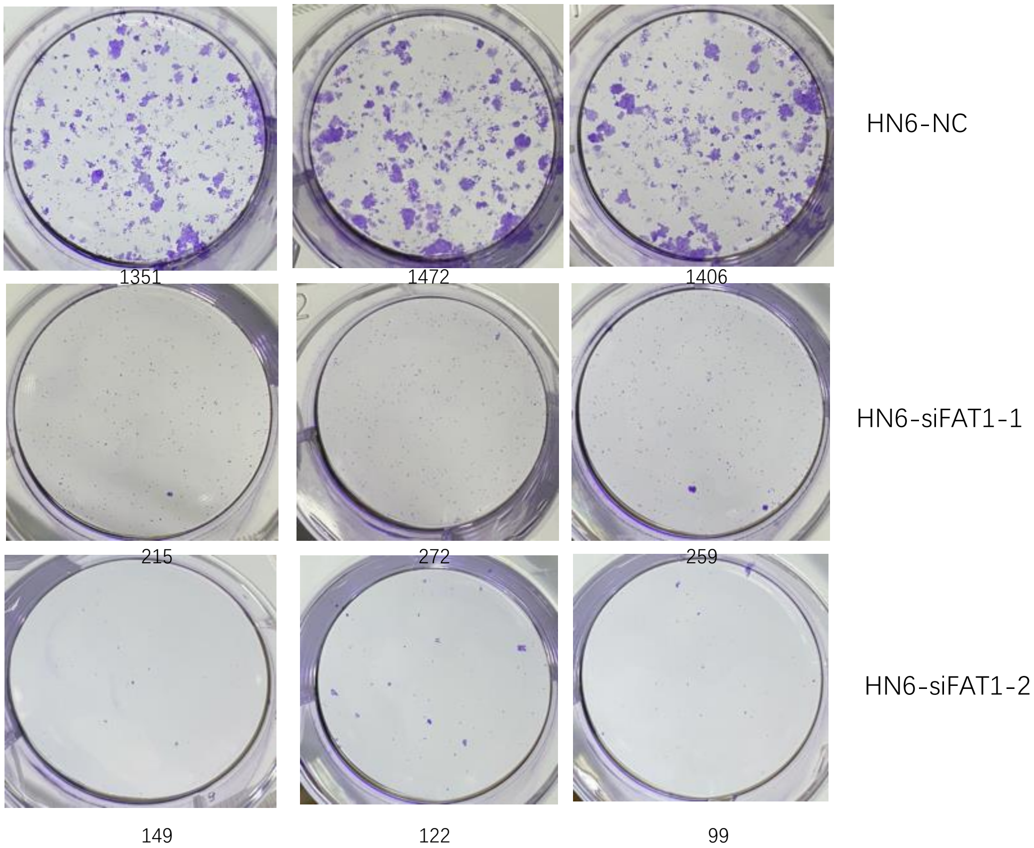

Fig4E

For HN6 cells

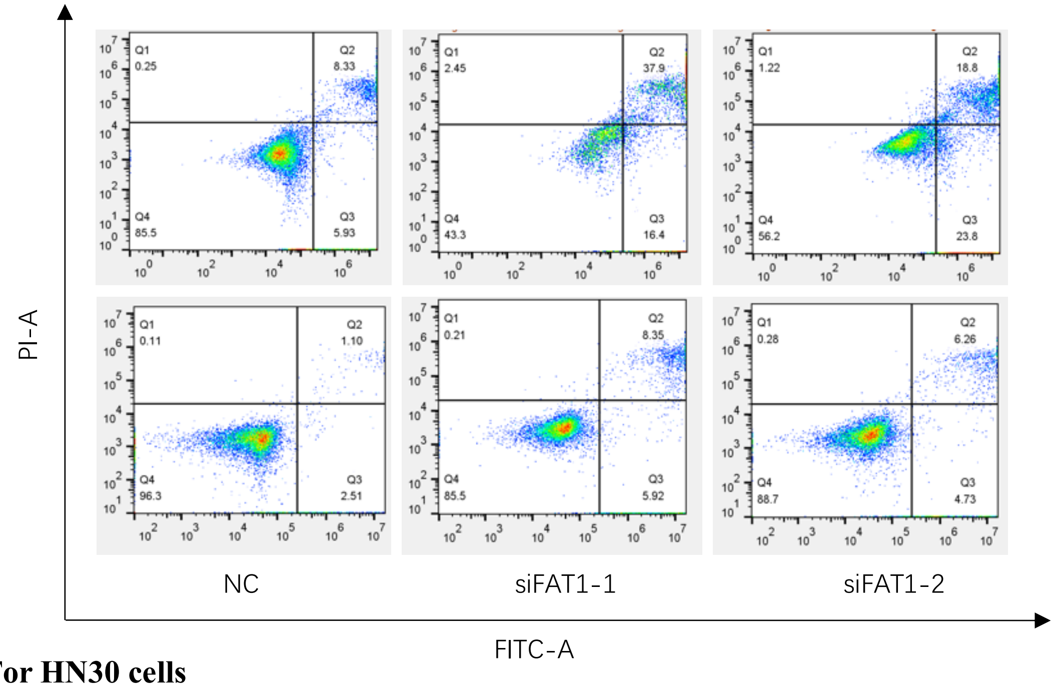

For HN30 cells

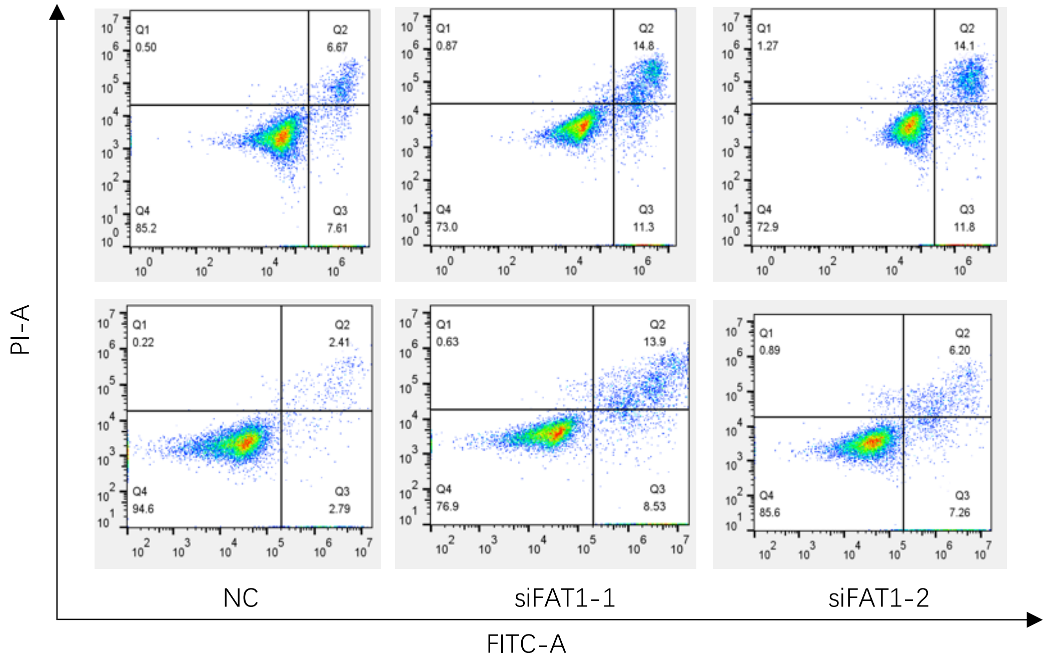

Fig4F

For HN6 cells

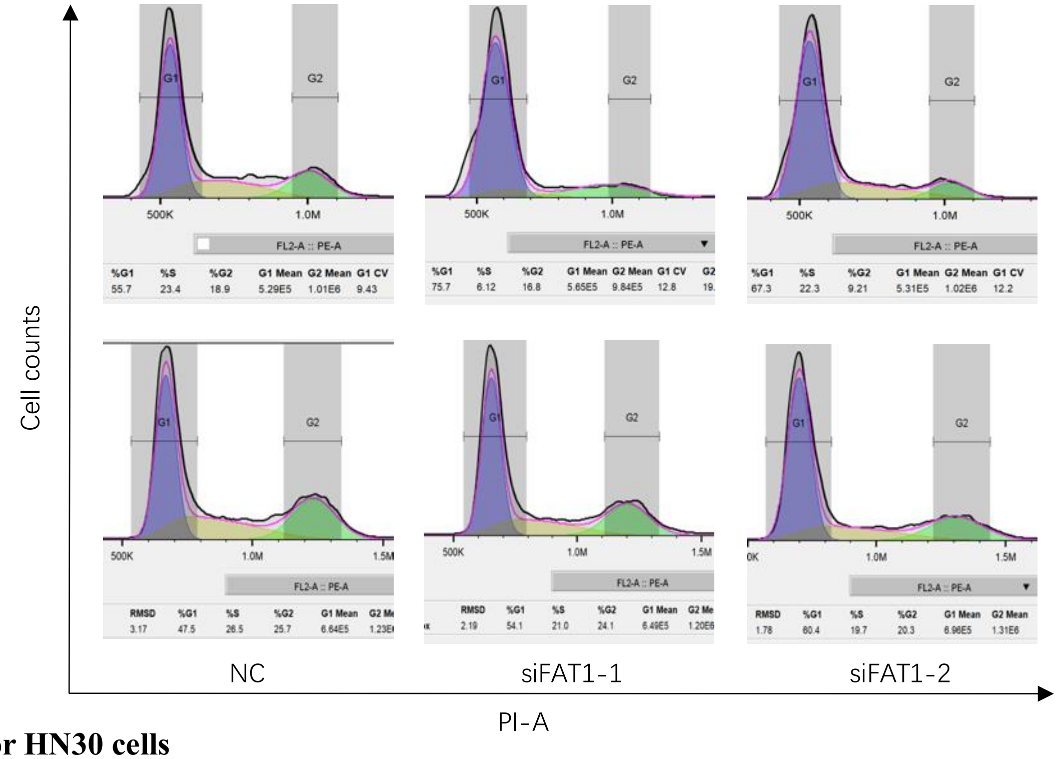

For HN30 cells

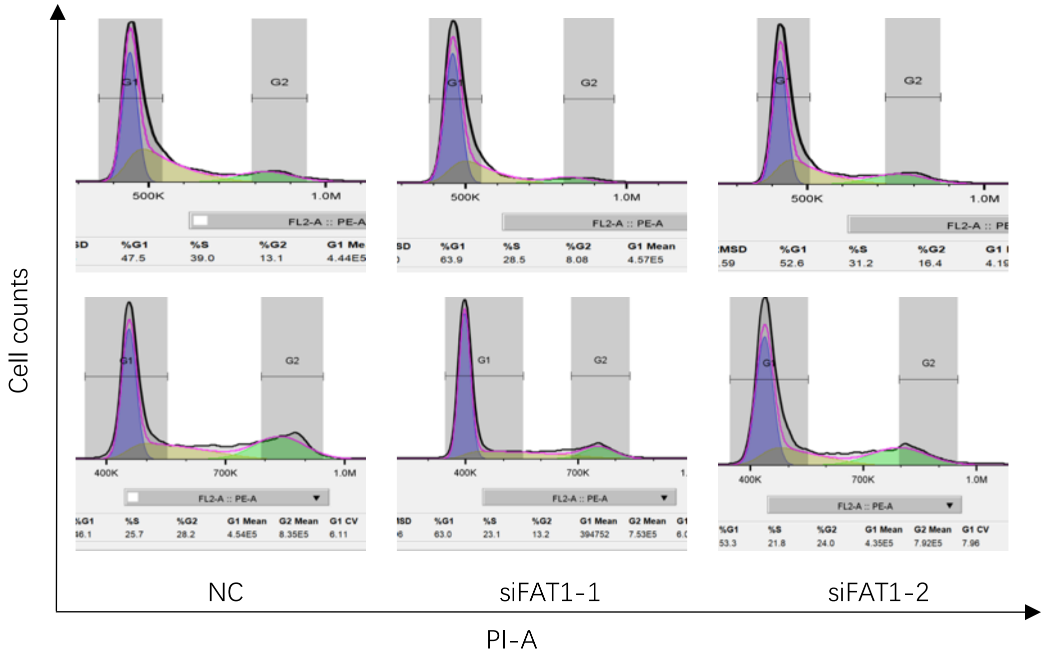

Fig4G

For HN30 cells

	NC	siRNA-1	siRNA-2
Core1	53	17	28
Core2	57	20	30
Core3	45	18	34

NC siRNA-1 siRNA-2

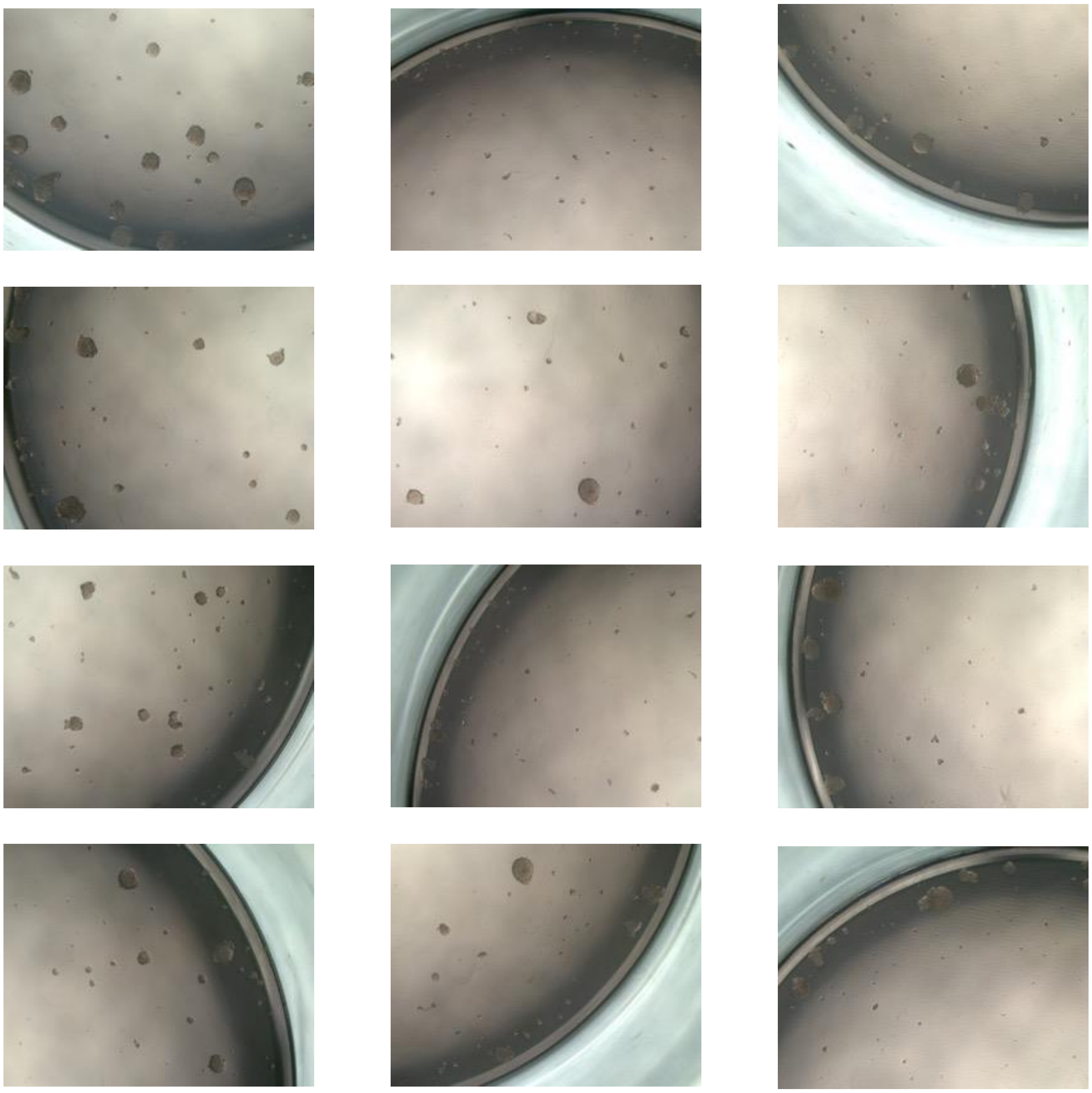

Photos of another independent repeat formation assay of HN30

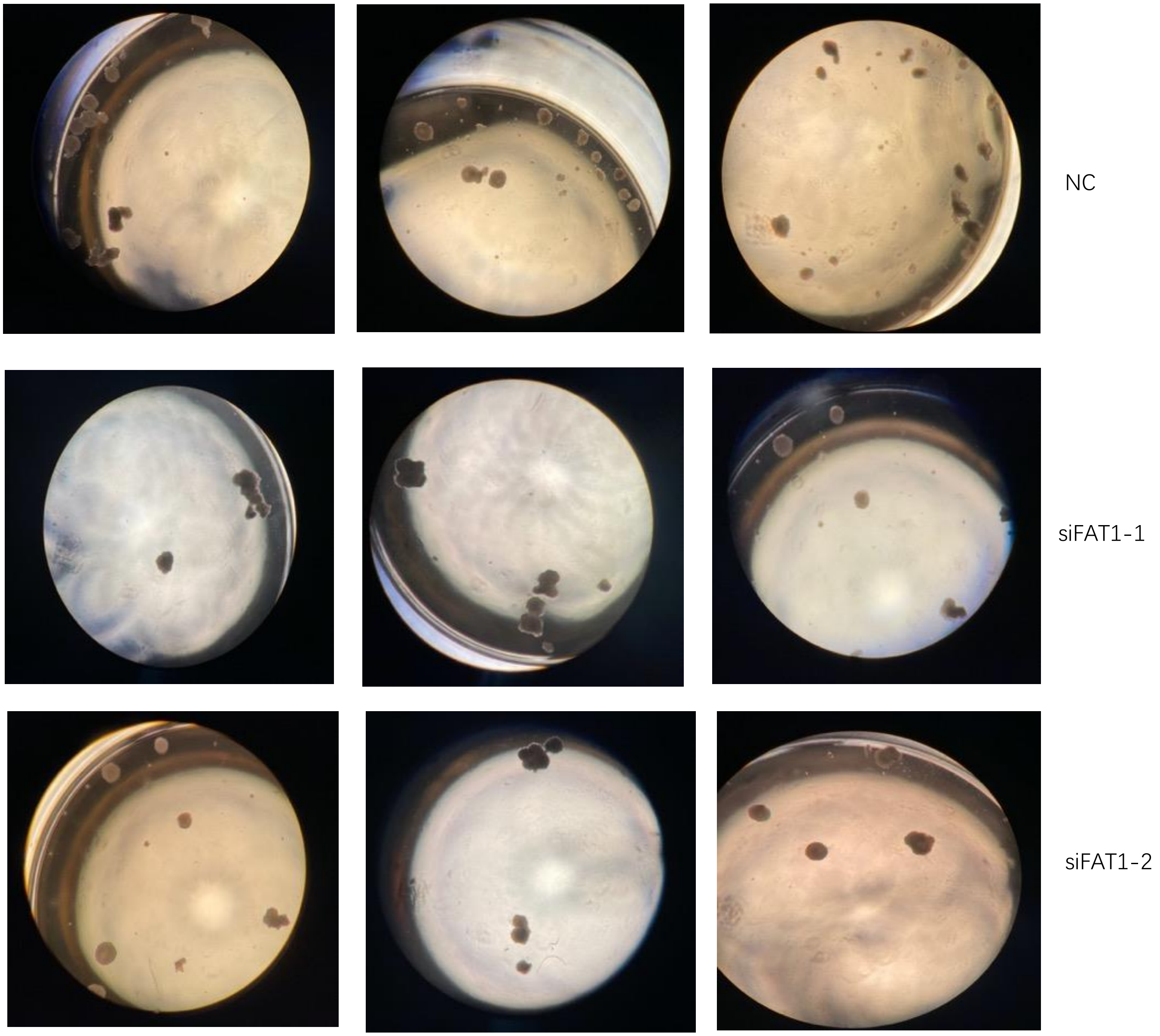

Fig5A

The full visual filed of transwell chambers of HN6 and HN30

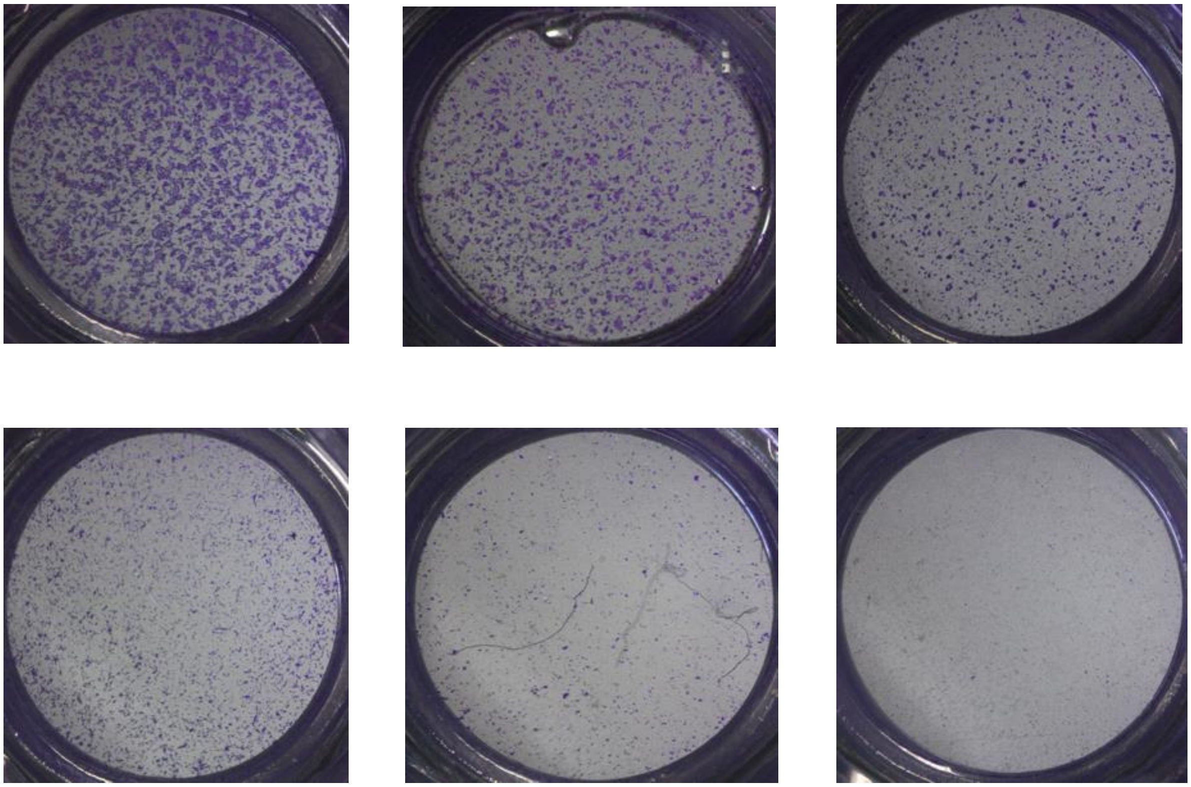

For HN6 cells

Number of migration cells

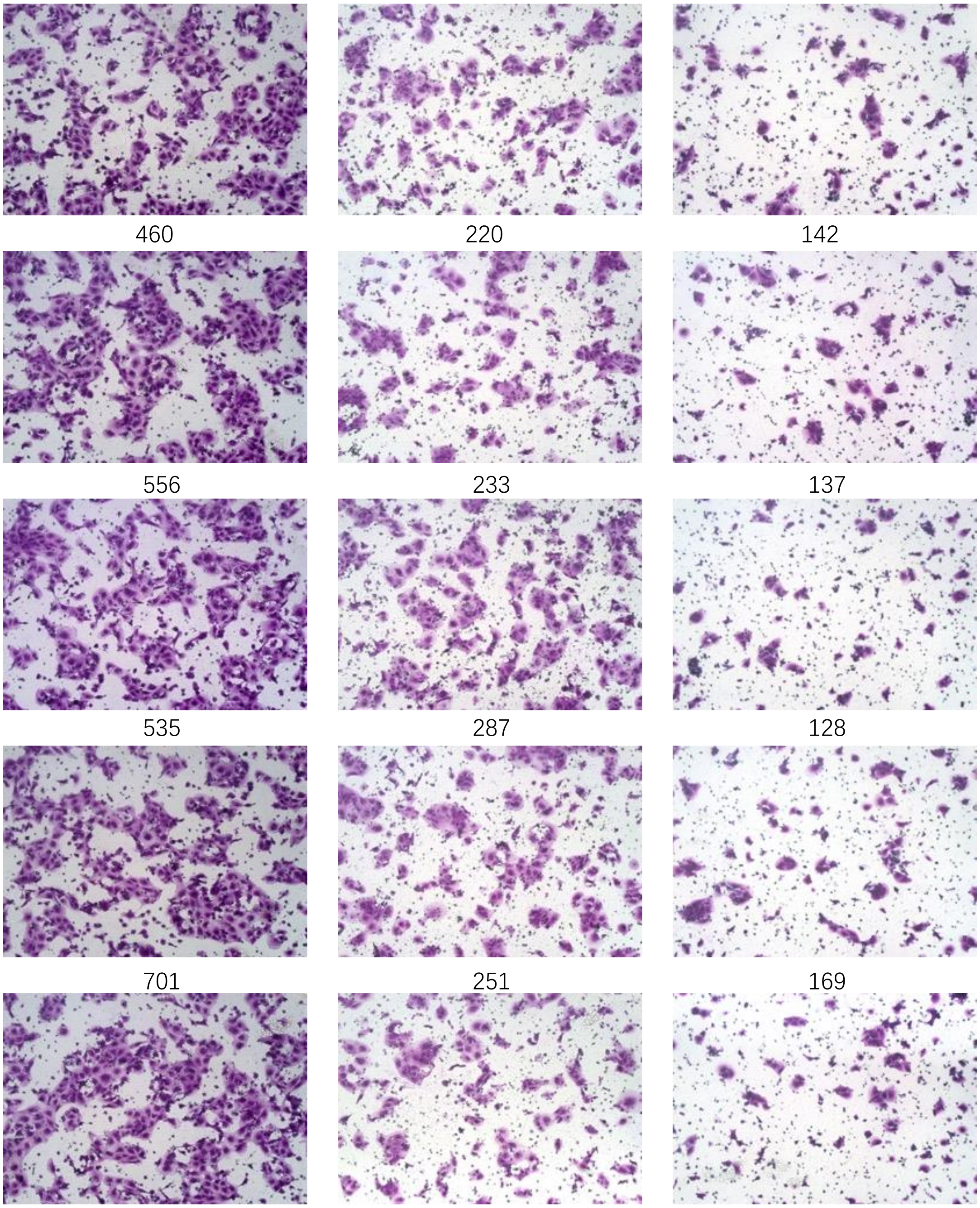

NC	siRNA-1	siRNA-2
460	220	142
556	233	137
535	287	128
701	251	169
680	196	171

For HN30 cells

Number of migration cells

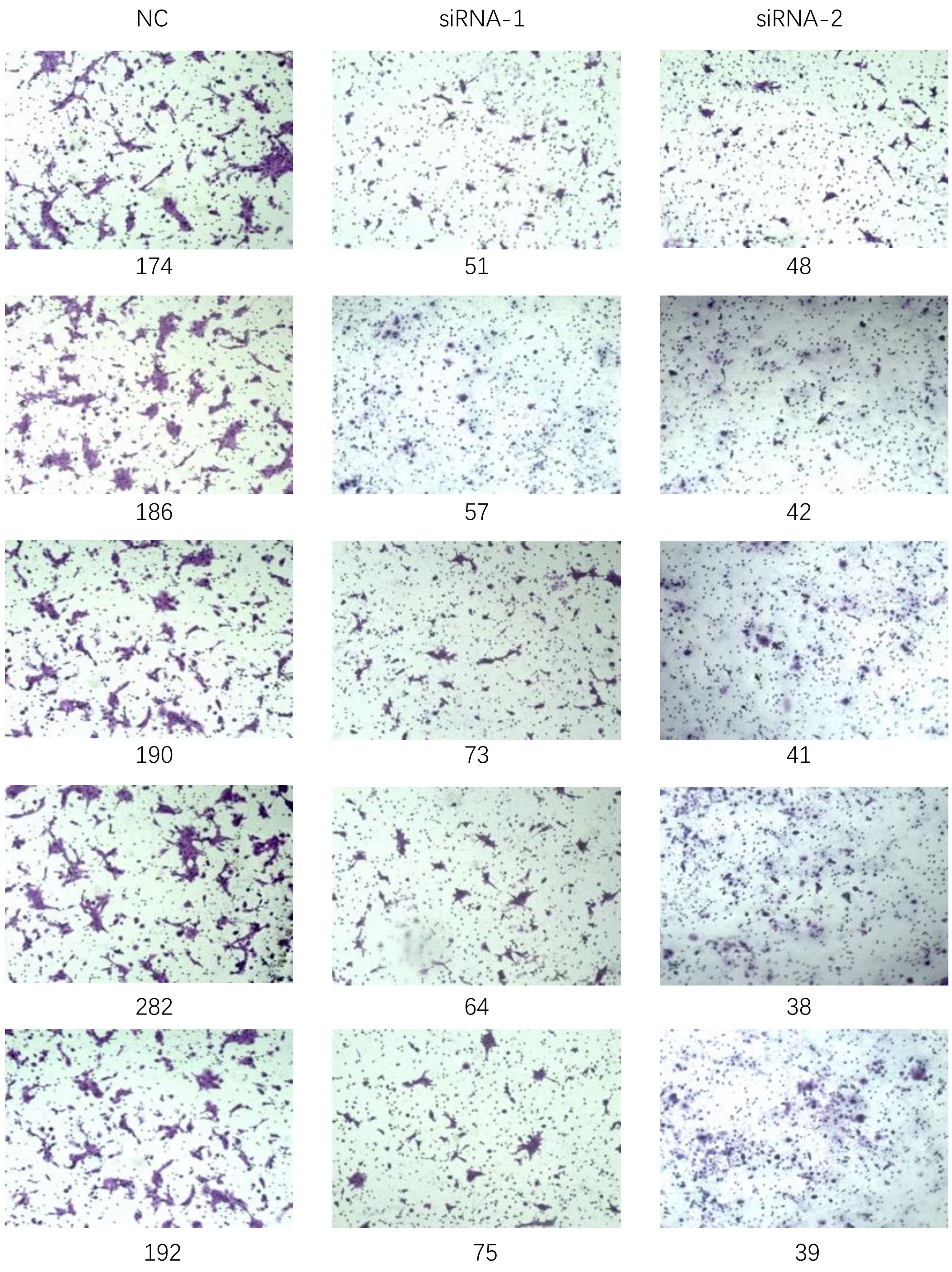

Fig 5B

For HN6 cells

Percentage of closure (calculated with image J software)

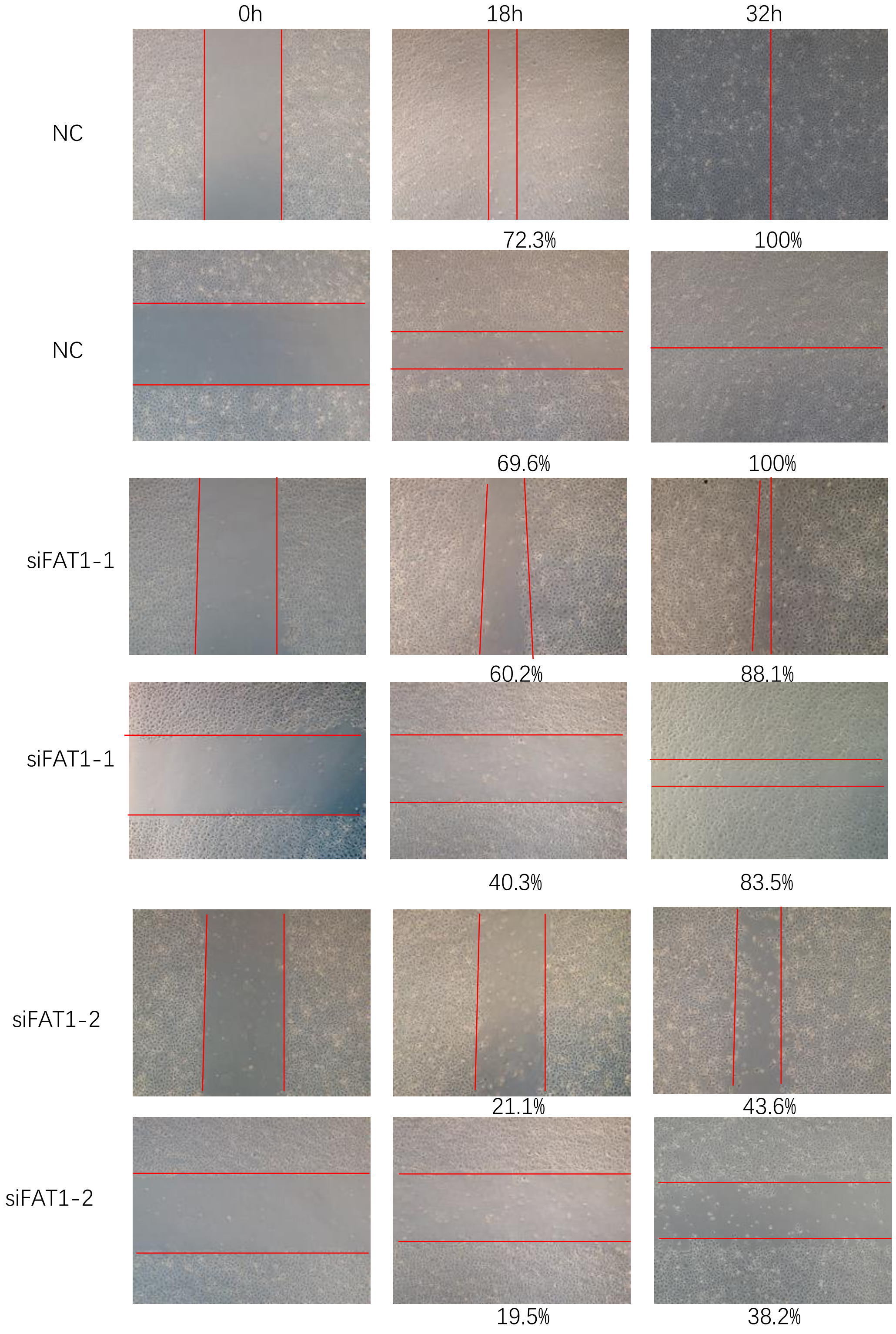

For HN30 cells

Percentage of closure (calculated with image J software)

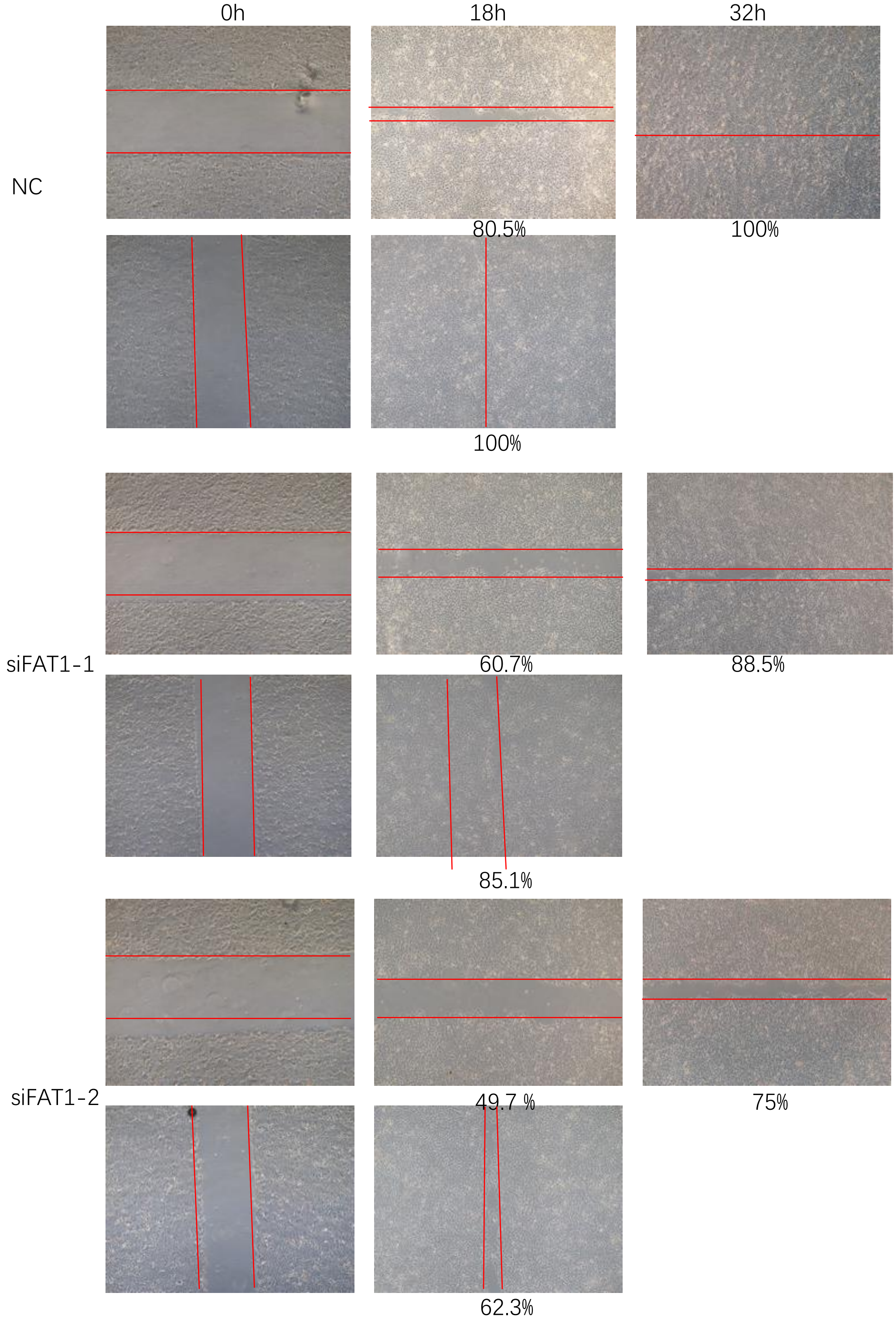

Supplement: Supplementary file 1 [file DataSheet_1.pdf]
